# *Point-of-Care* Autofluorescence Imaging for Real-Time Sampling and Treatment Guidance of Bioburden in Chronic Wounds: *First-in-Human Results*

**DOI:** 10.1371/journal.pone.0116623

**Published:** 2015-03-19

**Authors:** Ralph S. DaCosta, Iris Kulbatski, Liis Lindvere-Teene, Danielle Starr, Kristina Blackmore, Jason I. Silver, Julie Opoku, Yichao Charlie Wu, Philip J. Medeiros, Wei Xu, Lizhen Xu, Brian C. Wilson, Cheryl Rosen, Ron Linden

**Affiliations:** 1 Princess Margaret Cancer Centre, University Health Network and University of Toronto, Toronto Medical Discovery Tower, 15th floor, 101 College St., Toronto, Ontario, M5G 1L7, Canada; 2 Techna Institute for Advancement of Technologies for Health, University Health Network, 100 College Street, Rm. 124, Toronto, Ontario, M5G 1L5, Canada; 3 Prosserman Center for Health Research, Lunenfeld-Tanenbaum Research Institute, Mount Sinai Hospital, 60 Murray Street, Box 18 Room 5–236, Toronto, Ontario, M5T 3L9, Canada; 4 Dermatology, Toronto Western Hospital, University Health Network, 399 Bathurst St., Toronto, Ontario, M5T 2S8, Canada; 5 The Judy Dan Treatment and Research Center, 555 Finch Avenue West, Toronto, Ontario, M2R 1N5, Canada; 6 Department of Biostatistics, University Health Network, 610 University Avenue, Toronto, Ontario, M5G 2M9, Canada; 7 Dalla Lana School of Public Health, University of Toronto, 155 College Street, 6th floor, Toronto, Ontario, M5T 3M7, Canada; The National Institute for Health Innovation, NEW ZEALAND

## Abstract

**Background:**

Traditionally, chronic wound infection is diagnosed by visual inspection under white light and microbiological sampling, which are subjective and suboptimal, respectively, thereby delaying diagnosis and treatment. To address this, we developed a novel handheld, fluorescence imaging device (PRODIGI) that enables non-contact, real-time, high-resolution visualization and differentiation of key pathogenic bacteria through their endogenous autofluorescence, as well as connective tissues in wounds.

**Methods and Findings:**

This was a two-part Phase I, single center, non-randomized trial of chronic wound patients (male and female, ≥18 years; UHN REB #09-0015-A for part 1; UHN REB #12-5003 for part 2; clinicaltrials.gov Identifier: NCT01378728 for part 1 and NCT01651845 for part 2). Part 1 (28 patients; 54% diabetic foot ulcers, 46% non-diabetic wounds) established the feasibility of autofluorescence imaging to accurately guide wound sampling, validated against blinded, gold standard swab-based microbiology. Part 2 (12 patients; 83.3% diabetic foot ulcers, 16.7% non-diabetic wounds) established the feasibility of autofluorescence imaging to guide wound treatment and quantitatively assess treatment response. We showed that PRODIGI can be used to guide and improve microbiological sampling and debridement of wounds in situ, enabling diagnosis, treatment guidance and response assessment in patients with chronic wounds. PRODIGI is safe, easy to use and integrates into the clinical workflow. Clinically significant bacterial burden can be detected in seconds, quantitatively tracked over days-to-months and their biodistribution mapped within the wound bed, periphery, and other remote areas.

**Conclusions:**

PRODIGI represents a technological advancement in wound sampling and treatment guidance for clinical wound care at the point-of-care.

**Trial Registration:**

ClinicalTrials.gov NCT01651845; ClinicalTrials.gov NCT01378728

## Introduction

Chronic wounds and their associated care are a burden to patients and health care systems worldwide [1–5]. Current best clinical practice in wound infection diagnosis relies on bedside assessment of clinical signs and symptoms (CSS), such as pain, purulent exudate, crusting, swelling, erythema, foul odour, friable granulation tissue, wound breakdown and heat [2,5,6]. This involves subjective, qualitative visual examination under standard room lighting. Despite some common criteria, wound types may present with different clinical signs of infection [7,8], causing variance in clinical judgement [2,9,10]. Prompt recognition of wound infection enables timely and suitable interventions [1,11–15], which is important for managing chronic wounds such as diabetic foot ulcers [16]. However, subclinical bacterial load cannot always be detected by CSS, especially in asymptomatic patients, thus delaying the onset of wound treatment at the early stage of infection [17].

Microbiological testing [1,18] of wound samples is often used to identify and quantify bacterial species, the latter of which may be both an objective quantitative indicator of infection and a predictive correlate of healing [7,19,20]. Quantitative deep tissue biopsy is the gold standard for wound sampling and infection diagnosis, but is not always used because it is invasive, painful, and expensive [21]. Semi-quantitative surface swabs are the most common sampling method due to minimized expense and pain [21,22], but there is no conclusive guideline for the most effective swabbing technique [23]. The Levine swabbing technique [1] is most commonly used, but is limited because it samples the center of the wound only, potentiating missed collection and identification of treatment-relevant bacteria at the wound periphery or other distant locations. Moreover, the correlation of swabs to deep tissue biopsies as a reference standard is controversial [24–26].

Microbiology reports contain useful information about microbial identities, antibiotic susceptibility, and semi-quantitative bacterial growth rates, but these data typically represent the bacterial load in the wound centre only, and often arrive 3–5 days later. By this time, the wound’s biology and burden will have changed [11,27,28], which can lead to suboptimal treatment [29], including insufficient/ineffective debridement and broad-spectrum antibiotic therapy that encourages the emergence of antibiotic-resistant bacteria, such as Methicillin-resistant Staphylococcus aureus (MRSA), C. difficile, and Vancomycin-resistant Enterococci (VRE) [30].

There is an unmet clinical need to improve the microbiological sampling and treatment of wound infections. To address this need, we developed a handheld portable imaging device that obtains white light (WL) and fluorescence (FL) images (or video) of normal skin and wounds in high-resolution and in real-time, which can be used at the point-of-care. The device, which is Health Canada approved for clinical testing, is called PRODIGI (**P**ortable **R**eal-time **O**ptical **D**etection **I**dentification and **G**uide for **I**ntervention) and is based on the principles of autofluorescence (AF) [31]. In this *first-in-human* study, we demonstrate the initial clinical use of PRODIGI for real-time imaging of AF signals from pathogenic bacteria and tissues within and around chronic wounds, without exogenous contrast agents. PRODIGI was evaluated during two single-center, non-randomized trials, each of approximately 6 months duration in diabetic foot ulcer patients. We demonstrate that PRODIGI: 1) provides image-guidance for tissue sampling, detecting clinically-significant levels of pathogenic bacteria and wound infection otherwise overlooked by conventional sampling and 2) provides image-guidance for wound treatment, accelerating wound closure compared with conventional therapies and quantitatively tracking long-term changes in bacterial bioburden and distribution in wounds. These initial clinical results raise concern that conventional wound sampling may be suboptimal and that current clinical protocols may require careful re-examination and revision.

## Methods

### Study Design

The protocol for this trial and supporting STARD checklist are available as supporting information; see S1 STARD Checklist and S1 Protocol. This clinical study involved human subjects. The study was conducted as two Phase I, single center, non-randomized trials of patients with chronic wounds receiving clinical care. The trials were approved by the University Health Network research ethics board (UHN REB #09–0015-A for part 1; UHN REB #12–5003 for part 2) and listed on *clinicaltrials*.*gov* (Identifier: NCT01378728 for part 1 and NCT01651845 for part 2). All participants provided written consent on an IRB approved Informed Consent Form (ICF). The form required patient name, date and signature. A translator’s information was also obtained when a translator was used. In addition, an ICF check-list was used to note the verbal interaction between the patient and the consentor with respect to information provided verbally, questions the patient asked and timing of obtaining consent relative to procedures being performed. The research ethics committee/IRB approved this consent procedure.

The purpose of part 1 was to establish the safety and feasibility of AF imaging to improve wound sampling by accurately detecting clinically-significant levels of pathogenic bacteria in chronic wound patients, compared to standard wound assessment validated against blinded, gold standard swab-based microbiology. Part 2 was conducted to demonstrate the feasibility of AF image guidance for wound treatment and quantitate treatment response longitudinally, compared to standard non-guided treatment over three phases each of 2 months.

### Study Variables, End Points and Outcomes

For conventional clinical wound assessment, the primary study variables were: 1) wound size; 2) CSS; 3) tissue necrosis; and 4) swab cultures. For WL and FL imaging, the primary study variables were: 1) wound size; 2) average intensities, size and localization of green and red FL; and 3) quantitative FL spectra. Variables were assessed for each wound longitudinally. The study endpoint measurements were wound size and bacterial load. Swab cultures were used to compare AF imaging with WL examination, to determine sensitivity, specificity and predictive value of FL imaging for detecting clinically-significant bacterial loads. Outcomes in part 1 included demonstrating: 1) PRODIGI’s safety and integration into the work flow; 2) PRODIGI’s effectiveness for detecting clinical levels of bacterial burden missed by conventional techniques; and 3) the correlation between AF signals and bacteria/tissues. Outcomes for part 2 included: 1) developing a clinical protocol for FL image-guided wound treatment; 2) demonstrating the therapeutic benefit of FL image-guidance in closing wounds faster than conventional treatment; and 3) demonstrating the feasibility of longitudinal treatment monitoring using FL imaging.

### Patient Population

**Fig 1:**
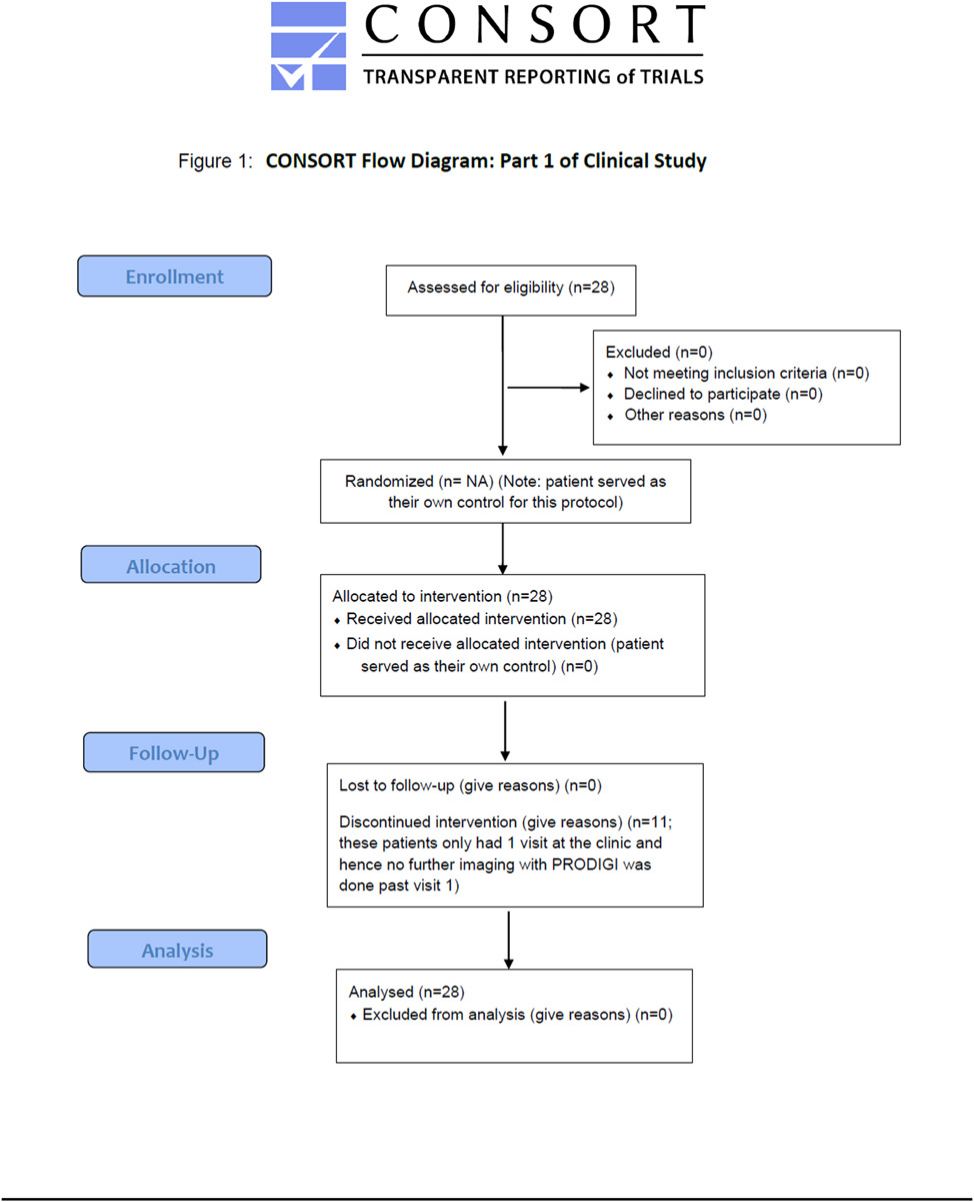
CONSORT Flow diagram for Part 1 of clinical study. Flow diagram of the progress through the phases of enrollment, allocation, follow-up, and data analysis for Part 1 of the clinical study.

**Fig 2:**
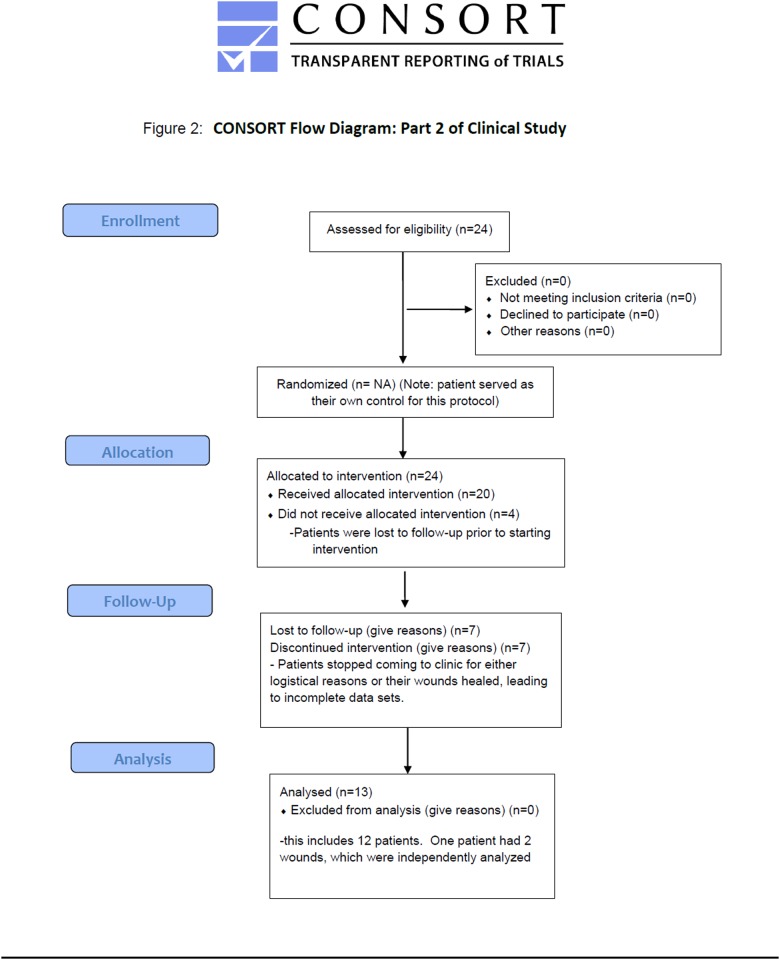
CONSORT Flow diagram for Part 2 of clinical study. Flow diagram of the progress through the phases of enrollment, allocation, follow-up, and data analysis for Part 2 of the clinical study.

Parts 1 and 2 were conducted at the Judy Dan Research and Treatment Center (JDRTC; Toronto, Canada). Males and females (≥ 18 years), diagnosed with chronic and/or acute wounds and with known or unknown infection status, who were already followed by JDRTC staff physicians were eligible. Most enrolled patients had non-healing neuropathic and neuro-ischemic diabetic foot ulcers, while others had non-diabetic wounds due to peripheral artery disease, venous stasis, soft-tissue and osteo-radionecrosis (54% diabetic foot ulcers, 46% non-diabetic wounds for part 1; 83.3% diabetic foot ulcers, 16.7% non-diabetic wounds for part 2). Exclusion criteria were: treatment with an investigational drug within 1 month before enrollment, contra-indication to routine wound care or monitoring, preexisting skin conditions (e.g. melanoma, psoriasis) in areas close to the wound(s), and an inability for written informed consent. Flow diagrams of the progress through the phases of enrollment, allocation, follow-up, and data analysis for Parts 1 and 2 of the clinical study are shown in Figs. 1 and 2.

### PRODIGI Imaging Device

**Fig 3:**
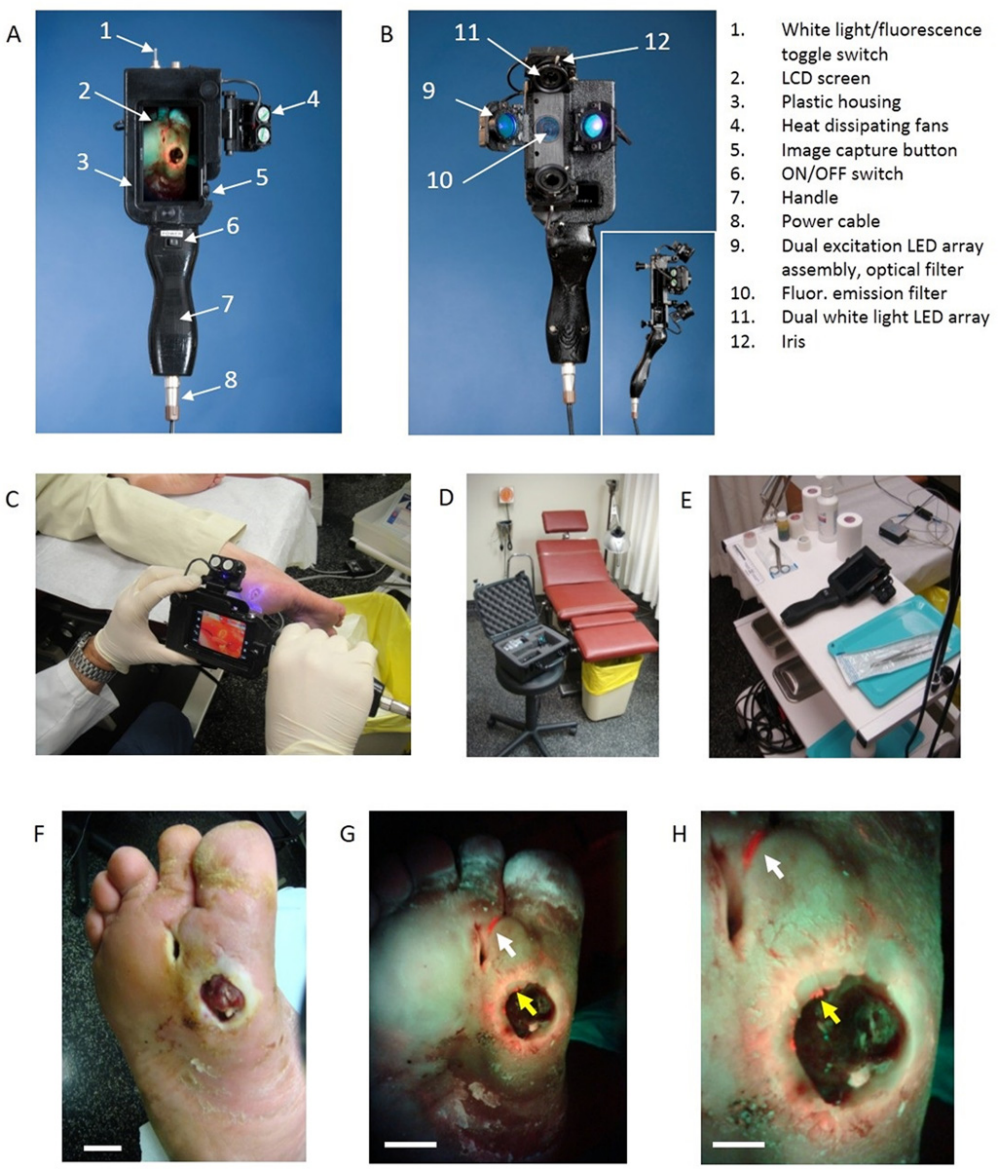
Photographs of the handheld prototype PRODIGI imaging device and its use for real-time autofluorescence imaging of bacterial load invisible by white light examination. *A*. Front view of PRODIGI showing wound fluorescence image displayed in real-time on the LCD screen in high definition. *B*. Back view of PRODIGI showing white light and 405 nm LED arrays providing illumination of the wound, while the fluorescence mission filter is placed in front of the CCD sensor. Inset shows side profile of the device. *C-E*. Photograph of PRODIGI device used to examine a diabetic foot ulcer with room lights on, in a hard shell carrying case in a typical wound clinic setting, and placed on typical wound care cart, respectively. Room lights are turned off for fluorescence imaging. *F*. PRODIGI white light image of type II diabetic foot ulcer in a 52 y old male patient. *G*. Corresponding AF image taken in < 1 sec showing bright red fluorescence of pathogenic bacteria in the wound periphery (yellow arrow) and in ‘off site’ areas (white arrow) away from the primary wound (confirmed by swab microbiology as mainly heavy growth S. aureus). Bacterial fluorescence appears red against a background of green fluorescence from connective tissues of the healthy skin, which provides anatomical context for localizing the bacteria within and around the wound. The bacterial regions were not seen under white-light visualization. *H*. A magnified view of *G*. showing S. aureus growing within the fissures of the wound periphery. Bright fluorescent ‘hot spots’ (yellow arrow) illustrate heterogeneity in the distribution of bioburden in the wound periphery. Fluorescence imaging allowed targeted swabbing of bacterial areas not possible by white light visualization. The heavy growth S. aureus growing in the off-site area was invisible by traditional clinical examination. *Scale bars*: *A*. *2 cm*, *B*. *2 cm*, *C*. *1 cm*.

PRODIGI (Fig. 3) is approved by the Canadian Standards Association and Health Canada as a Class II medical device for clinical investigational testing (Health Canada Investigational Testing Authorization #16114) and has been tested pre-clinically [32]. The PRODIGI prototype consists of a low-cost consumer-grade Super HAD CCD sensor-based camera (Model DSC-T900, Sony Corp., Japan) with a 35–140 mm equivalent 4x zoom lens housed in a plastic body and powered by rechargeable batteries (Fig. 3A, B). It collects high-resolution 12.1 megapixel color WL and AF images (or videos), which are displayed in RGB format on a 3.5-inch touch-sensitive color LCD screen (Fig. 3B). Broadband white LEDs, electrically powered by a standard AC125V source, provide illumination during WL imaging, while two monochromatic violet-blue (λ_exc_ = 405 ± 20 nm) LED arrays (Model LZ4, LedEngin, San Jose CA) provide 4 Watt excitation light power during FL imaging (bright, uniform illumination area ~700 cm^2^ at 10 cm distance from skin surface). WL and FL images are detected by a high-sensitivity CCD sensor mounted, with a dual band FL filter (λ_emiss_ = 500–550 and 590–690 nm) (Chroma Technologies Corp, VT, USA) in front of the camera lens to block excitation light reflected from the skin surface. Tissue and bacteria AF are spectrally separated by the emission filter and displayed as a composite RGB image without image-processing or color-correction (Fig. 3F, G, H), allowing the user to see the bacteria location and distribution within the anatomical context of the wound and body site (Fig. 3F, G, H).

### Patient Assessment and Clinical Procedures

Patients underwent complete physical, medical history, and wound assessments. Wounds were prepared by standard methods, assessed for CSS using the CSS Checklist (CSSC) [33], and categorized as infected or non-infected. For chronic diabetic wounds, the type and amount of wound bed tissue was measured using the necrotic tissue subscale of the Pressure Sore Status Tool (PSST) [34]. Necrotic tissue was defined as yellow, tan, brown, gray or black tissue in the wound bed after surface cleansing with saline-moistened gauze [35]. Wound size and depth were measured at each visit. Surface area was measured by tracing the wound edge on WL images (the line dividing healed and unhealed portions). Wound area was considered a valid marker of wound size [36]. Wound depth was measured using a swab placed in the deepest portion of the wound.

In part 1, high resolution WL PRODIGI images were taken of every wound at each visit. A disposable length calibration scale (sticker) was placed near the wound during WL and FL imaging to track patient ID and date. A clinician marked the locations of suspected clinically significant bacterial load on printed WL images. To preserve bacterial characteristics on the tissue, no swab was taken until completion of subsequent FL imaging. This process took 1–2 min per wound, and subsequent FL imaging took 1–2 min per wound. The location(s) of positive red and/or green AF were marked on printed images. The clinician swabbed each suspicious marked area using the Levine sampling method [1] and swabs were sent for blinded microbiology testing. A detailed description of swabbing, specimen collection and microbiology can be found below. Patients were treated and discharged according to standard protocols. FL spectroscopy, as described below, was used in some cases to characterize AF areas in/around the wound. Spectra were compared on a location basis with microbiology results. A complete data file for each patient’s visit (CSS, WL and FL images, spectroscopy and microbiology) were stored in an electronic database according to Good Clinical Practice guidelines.

Part 2 consisted of three sequential 2-month arms: non-guided treatment (control), FL-guided treatment and non-guided treatment (control). In the first 2-month phase, wounds were assessed weekly by CSS and then treated at the discretion of the clinical team using best practice methods (ultrasonic and/or scalpel wound debridement, topical/systemic antibiotics, saline wash, dry or anti-microbial dressings or iodine). Corresponding WL and FL images were taken of each wound pre- and post-treatment as described previously. We chose 2 month evaluation periods based on established clinical data for venous leg ulcers showing that this is sufficient to detect a reliable and meaningful change in wound area, as a predictive indicator of healing [37].

Wound swabs were collected by FL guidance. Clinicians were blinded to FL images during this first (control) phase. During the subsequent 2 month phase, wound assessment was performed normally but clinicians were shown FL images of the wound during treatment. During the final 2 month phase, WL and FL imaging were performed and swabs were collected, with clinician blinding to the FL results during treatment delivery.

Importantly, while the clinicians understood and could remember the meaning and characteristics of the red and green fluorescence signals, respectively, blinding them during treatment delivery in the control periods was possible because the fluorescence results for each wound examination and each patient were different. Thus, in the absence of real-time fluorescence guidance during wound treatment, previous knowledge of fluorescence characteristics did not substantively influence the treatment decisions during the control periods.

WL and FL images were also taken after each treatment to analyze wound area. Four blinded, trained clinical and/or research staff members independently measured the average wound size on WL images using digital tracing (MATLAB v.7.9.0, The MathWorks, Massachusetts, USA). The observers measured the wounds in separate sessions with a minimum of 7 days between sessions to minimize memory effect. An adhesive scale bar placed adjacent to the wound during imaging provided accurate length calibration within ±0.5 mm.

### Fluorescence Imaging Procedures

Room lights remained on during WL imaging, but were turned off during FL imaging. WL and FL images were collected with PRODIGI held 10–15 cm from the wound. All imaging parameters (distance, exposure time, ISO setting, white balance, etc.) were kept constant between visits. For distances less than 5 cm from a wound (small diameter wounds), the camera’s built-in macro mode was used. Automatic focusing allowed rapid (~1s) image acquisition. Images (or video) were captured in real-time and stored on the camera’s memory card. Switching between WL and FL modes was instantaneous using a built in ‘toggle switch’ (Fig. 3A). Devices were decontaminated between uses with 70% ethyl alcohol. To judge how the technology integrated into routine workflows, the research team documented: 1) adverse events; 2) average time for FL imaging; 3) placement of the device; and 4) survey results of clinical users after each procedure.

### Swabbing, Specimen Collection and Microbiology

The Levine technique [1] was used to aseptically swab wounds for confirmation of bacterial presence, species typing, Gram signing, antibiograms and semi-quantitative bacterial load. Neither antiseptic solution nor lavage was introduced to the wound before swabbing, to ensure accurate collection of fluorescing bacteria [31]. Swabs were placed in contact with the specified area and held for at least 5 s, then placed in sterile transport tubes containing modified Stuart’s medium, immediately transported to the microbiology laboratory and processed within 1–2 h of acquisition. Cultures were performed by standard procedures [1,38]. Microbiological assessment included semi-quantitative colony estimates, and scoring for occasional, light, moderate or heavy growth. A heavy growth bacteria from the swab culture can often be equated to approximately 10^5^ colony-forming units of bacteria per gram of tissue [39].

### Fluorescence Spectroscopy

A custom-built portable spectroscopy device (USB2000+, Ocean Optics, Dunedin, Florida, USA), with 0.35 nm spectral resolution and sensitivity between 350–725 nm, was used to characterize tissue and bacterial AF, particularly the relative intensity and contribution of the green and red FL to the total signal. Spectra were collected using PRODIGI’s 405 nm excitation illumination. An optical fiber probe with a long pass 450 nm emission filter was used to collect the FL signal (490–800 nm). Ten FL spectra were averaged from each measurement site that was suspicious for bacterial infection. The locations where spectra were obtained were documented on digital WL images for location-based comparison with microbiology.

### Image Analysis

WL and AF images were transferred to a laptop. Regions of interest (ROIs) were identified from individual 1024x1024 pixel FL images of each wound at each clinic visit. RGB images were separated into individual channels. The green and red channels of the RGB image were representative of the true tissue and bacterial AF signals detected *in vivo*. To quantify bacterial levels from individual FL images, the following image processing procedures were used. Briefly, individual green and red image channels from each RGB image were converted to greyscale (the blue channel was not used) and pixels whose greyscale intensity was above a given histogram threshold (selected to reduce the background noise of the raw image) were counted. A red color mask for red FL bacteria was created by finding the local maxima in the color range 100–255 greyscale. Then, an inverted green color mask was used to remove the green FL. All pixels with red FL (above the histogram threshold) were binarized and the sum of all “1” pixels was calculated. This was repeated for the green channel of each image. These data gave an estimate of the amount of red (or green) bacteria in each image. The number of FL pixels was converted into a more useful pixel area measure (cm^2^) using the adhesive length calibration stickers, thereby providing the total amount of fluorescent bacteria as an area measurement.

### Statistical Analysis

Mixed statistical models were used, with wound size and semi-quantitative microbiology colony estimates as outcomes. The FL intensity (total or ROI) for green and red channels, bacteriology results and the imaging time-point were used as explanatory variables. Wounds were categorized as bacteria-positive (measured by microbiology as occasional light, moderate or heavy growth) or bacteria-negative, and were examined for differences in CSS using Fisher’s exact tests, and differences in microbial load and species diversity using t-tests for independent groups. An alpha level of 0.05 (two-tailed) was employed. Sensitivity and specificity for detecting bacteria CSS or FL was calculated per-wound and per-patient (to assess ability to detect *and* correctly diagnose bacterial contamination). Among all the 133 measurements, only four of them are missing or indeterminate. These were tested to be missing at random and the linear mixed effect model is suitable for missing random data. We excluded these four missing measures from our analysis. There were no outliers detected in the dataset.

Categorical variables were described using counts and proportions. Exact binomial confidence intervals for proportions were calculated when assuming independence of observations. In most cases, generalized estimating equations (GEE) logistic regression models were used to estimate and compare proportions when accounting for clustering. GEE logistic models could not be used to estimate sensitivity or specificity for FL, or to compare between FL and WL, because all swabs were positive under FL. In those cases, the SAS macro %CLUSTPRO [40] was used, with exact confidence intervals only for estimation. Statistical analysis was carried out using SAS v 9.2 [40]. No adjustments were made for multiple tests. We used a bootstrap resampling algorithm to test the robustness of the estimation. The bootstrap confidence intervals (CIs) were calculated and compared with the model based CIs. We found that the parameter estimations are quite robust. In addition, the normality of distribution of the average wound area was tested using Shapiro-Wilk test with p-value calculated to be greater than 0.05, which suggested the normal distribution of the data. We also calculated the robust Cronbach’s alpha value to assess the agreement of measures. The alpha value is 0.997 using “cronbach” function of R package “coefficient alpha” and this suggested that there is excellent agreement between different measures.

To compare the variation in measurements of wound areas between images taken under WL and FL, the following steps were taken. For each wound imaged, the area measurements made by both assessors were combined to create a summary measurement of variation for the WL image and another for the FL image. The difference between the 2 summary measurements was taken. To test for a difference in the levels of variation, an intercept-only mixed linear regression model was implemented to account for repeated differences calculated for wounds that were examined more than once. To test whether the mean difference in variations is different from zero, the intercept equals zero test was used. Ages were described with medians and ranges.

Statistical significance of the sensitivities and specificities of CSS and AF imaging was calculated using McNemar’s test. For comparison among groups, Fisher’s exact test was used. Accuracy was defined as the percentage of all patients or wounds in which AF imaging correctly predicted the presence or absence of clinically-significant pathogenic bacteria confirmed by swab-based microbiology testing. P ≤ 0.001 was considered significant.

In part 2, correlation between change in average wound area and FL image-guided treatment was tested using Pearson correlation coefficients. Assessing changes in wound area between the first control, second FL image-guided, and third control periods were performed using a linear mixed effect model [41]. P ≤ 0.05 was considered significant. The number of measurements, measurement dates and the starting date of the guided period varied between patients. Mixed model regression was used to model the whole process together on the repeated measures of the wound area. The slopes of each period were calculated and the differences between them assessed.

## Results

### Participants

In part 1, 28 patients were enrolled and followed serially during clinic visits over approximately 6 months. Patients included 5 females (17.9%) (mean age 55 y; median 44 y; range 22–89) and 23 males (82.1%) (mean age 56.6 y; median 55 y; range 20–91). Across all 28 patients, the mean age was 56.3, the median 52.2 y, and the range was 20–91 y. A total of 48 wounds were tracked during each visit with swab culture tests performed at each visit. Most patients had a single wound with 13 (27%) having more than one wound. The wounds assessed included 35 foot and ankle (73%), 8 leg (17%), 3 hand (6%), 1 breast (2%) and 1 mouth (2%) wound. Among the 28 participants, 13 (43%) were seen only once, 12 (40%) were seen between 2 and 5 times and 5 (17%) were seen between 6 and 12 times.

Part 2 involved a separate patient cohort. A total of 12 patients with chronic wounds were enrolled and followed for up to 6 months: 2 females (16.7%) (mean age 75.5 y; median 75.5 y; range 71–80 y) and 10 males (83.3%) (mean age 65.5 y; median 65 y; range 50–81 y). Across all 12 patients, the mean age was 67.2 y, the median was 68 y, and the range was 50–81 y. A total of 13 wounds were evaluated. Most patients had a single wound with 1 patient (8.3%) having two wounds. The wounds assessed included 12 foot and ankle wounds. Seven patients (58.3%) were seen between 7 and 10 times, 4 (33.3%) between 11 and 14 times and 2 (16.7%) between 15 and 19 times. Multiple visits allowed longitudinal comparison between wounds treated without and with FL image-guidance over 3 periods, each lasting 2 months.

### PRODIGI provides immediate visualization of bacterial load invisible by white light

Tissue AF produced by endogenous collagen or elastin in the skin appeared as green FL, and clinically-relevant bacterial colonies (e.g. Staphylococcus aureus) appeared as red FL (caused by endogenous porphyrins [42–44]). Some bacteria (e.g. Pseudomonus aeruginosa) produced a blue-green signal, due to siderophores/pyoverdins [45,46], which was differentiated spectrally and texturally from dermis AF using image analysis software. WL and FL images were collected in less than 1 second by the high-sensitivity CCD sensor mounted with a dual band FL filter (λ_emiss_ = 500–550 and 590–690 nm) (Chroma Technologies Corp, VT, USA). The CCD image sensor was sensitive across a broad wavelength range of ~300–800 nm. PRODIGI integrated easily into the routine clinical work flow (Fig. 3C–E). By combining tissue FL with bacterial FL in a single composite image, the clinician instantly visualized the distribution and extent of the bacterial load within the anatomical context of the wound and body site (Fig. 3F, G). Typically, FL imaging added approximately 1–3 minutes/patient to the wound assessment routine, depending on the number of wounds and the duration of FL-guided swabbing. No adverse events were noted.

### Autofluorescence accurately detects bioburden in the wound bed, periphery and off site

We compared the accuracy of identifying clinically-significant bacterial load for AF image-guided swabbing versus WL. Validation was performed using semi-quantitative microbiological tests from swabs obtained by either WL or FL image-guidance. Results were reported on a per-swab basis as: i) occasional, light, moderate or heavy bacterial growth [1,47]; ii) identification of clinically-significant microbes; and iii) susceptibility antibiograms by a blinded independent microbiology laboratory. A total of 490 swabs were collected by clinical staff from 48 wounds in 28 patients and from additional surrounding tissue suspected of being colonized or infected. Swab collection was guided either by WL CSS examination or AF imaging, and sent for microbiological analysis. Of the 490 swabs, 36.9% were taken from wound beds, 30.2% from wound peripheries and 32.9% from “off-site” areas. Accuracy was defined as incidences in which either methodology (CSS with WL imaging or AF imaging alone) correctly judged that a microbial swab was required. Importantly, our previous preclinical analysis showed that pathogenic bacterial AF quantitatively correlates with wound bacterial load *in vivo* [32]. Of the 48 wounds, 7 produced unremarkable FL. Accordingly, 41 wounds (85.4%) expressed some degree of visible FL, of which 34 (82.9%) expressed red FL and 19 (55.9%) expressed green FL. Of the 41 fluorescent wounds, 12 (29.3%) showed both red and green bacterial FL, 22 (53.7%) expressed only red bacterial FL and 7 (17.1%) expressed only green bacterial FL.

**Fig 4:**
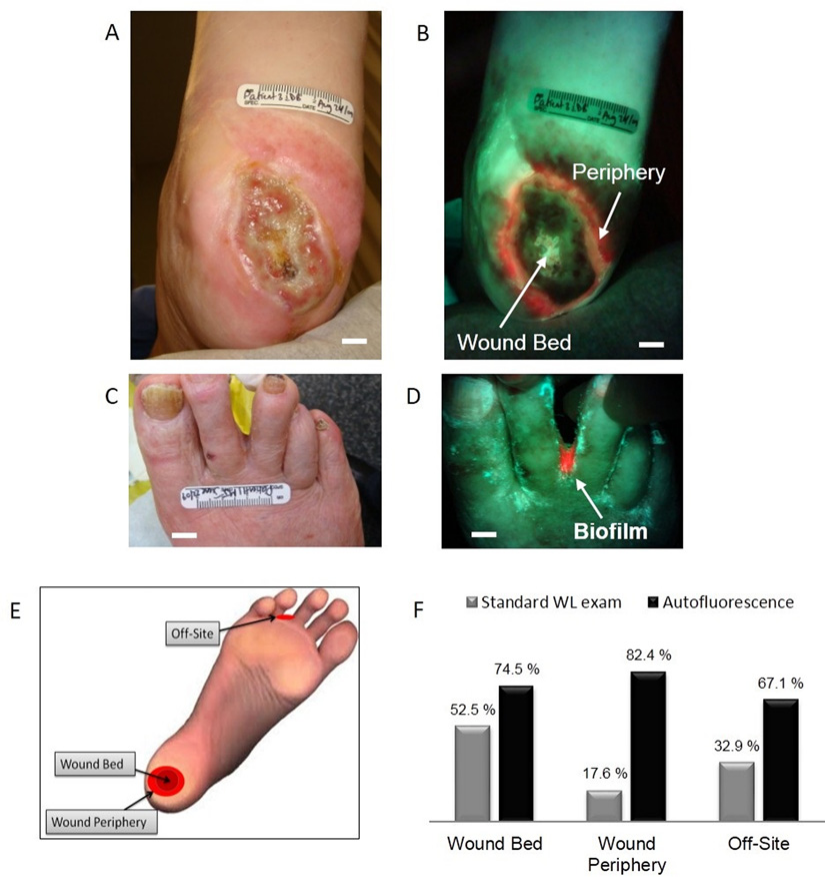
Autofluorescence detection of clinically-significant bacterial load in wound periphery and off-site areas. *A*. White light image of a type II diabetic foot ulcer in a 78 y old female. *B*. Corresponding AF image showing heavy growth of S. aureus in the wound periphery missed by white light imaging. *C*. White light shows unremarkable areas between toes, while in *D*. the corresponding AF imaging detected bacterial biofilm, confirmed by microbiology. *E*. Schematic illustrates different wound locations where fluorescence imaging detected clinically significant bacterial load. *F*. Comparison of accuracy for correctly detecting clinically-significant bioburden between standard WL and AF imaging in wound bed, wound periphery and off-site areas. *Scale bars*: *A*,*B*. *1 cm; C*. *2 cm*, *D*. *1 cm*.

We also examined the location of bacterial FL. Of the 41 fluorescent wounds, bacterial FL was present within the wound bed in 32 cases (78.0%) and within the periphery in 29 (70.7%) cases. Twenty-six unique and unexpected offsite locations of bacterial FL were observed in 18 of 28 patients (64.3%), which were missed by WL examination. AF accurately detected clinically-significant bioburden in surrounding locations close to wounds 67.1% of the time, where traditional clinical methods did not examine or swab (Fig. 4F). In 15 (36.6%) of the 41 FL-positive wounds, FL was present in both the wound bed and the periphery. Two (4.9%) of the 41 wounds had FL only at an arbitrary off-site location away from the wound, with another 2 (4.9%) having FL in both the wound bed and at an arbitrary off-site location.

An accuracy comparison was performed between WL and AF for detecting clinically-significant bioburden in the wound bed, wound periphery and in off-site areas. In the wound bed, AF correctly detected 74.5% of wounds with clinically significant bioburden compared with 52.5% for WL inspection (p = 0.003). In the wound periphery and off-site areas, standard practice would not have assessed these areas during wound examination, while PRODIGI accurately detected clinically significant bioburden 82.4% of the time in the wound periphery (p < 0.001) and 67.1% in other areas (p = 0.006) (Fig. 4F.). WL examination was correct 17.6% of the time in peripheries and 32.9% in other areas (p < 0.001 and p = 0.006, respectively). Thus, by not assessing these areas at all, standard practice failed to detect 82.4% and 67.1% of clinically significant bacterial load in the periphery and off-site regions, respectively (Fig. 4A–F).

Moreover, longitudinal FL imaging showed that 90% of patients would have been sent home at least once during the care cycle with a clinically-significant bacterial load if assessed by CSS and WL (95% confidence interval: 81.0%, 96.0%). Overall, CSS with WL examination had a positive predictive value (PPV) of 94.5% *vs*. 74.5% for AF imaging (p < 0.001) but the sensitivity of standard methods was only 14.2% (p < 0.001). The ratio of true positives from AF imaging to standard methods was 365/52 = 7.0. The overall accuracy of judging the presence of clinically-significant bacterial load in chronic wounds for AF was 74.5% *vs*. 35.5% (p < 0.001) for CSS with WL, based on swab results.

### PRODIGI spectrally differentiates P. aeruginosa from other pathogenic species in situ

**Fig 5:**
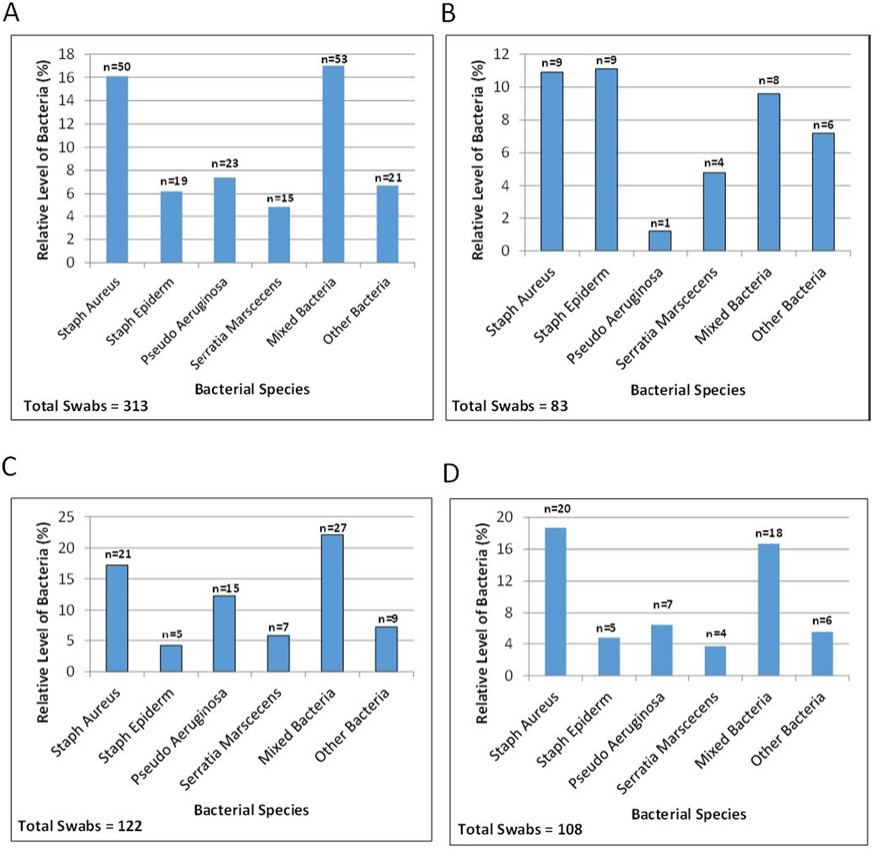
Wound swabs taken under autofluorescence imaging guidance, indicating detected polymicrobial species prevalence. The percentage of bacteria for each species are plotted for *A*. all swabs, *B*. swabs obtained from the wound bed, *C*. from the wound periphery and *D*. ‘off-site’ areas away from the primary wound.

Using λ_exc_ = 405 ± 20 nm and λ_emiss_ = 500–550 nm, 590–690 nm, PRODIGI detected AF signals of S. aureus, S. epidermidis, P. aeruginosa, Candida, S. marcescens, S. viridans, Diptheroid bacilli, S. pyogenes (β-hemolytic streptococci), Enterobacter, and Enterococci, and methicillin-resistant S. aureus (MRSA) and β-hemolytic streptococci, as verified by swab cultures. Swabs obtained by PRODIGI image guidance detected polymicrobial species, with variations in the relative levels of bacterial species across all swabs collected and from the wound bed, wound periphery, and off-site areas (Fig. 5A–D). Bacterial FL signals appeared bluish-green and red mainly *in vivo*, differing significantly in spectral characteristics from connective tissues and blood which appeared green and dark red, respectively. PRODIGI could differentiate bluish-green fluorescent P. aeruginosa from other red fluorescent bacterial species *in situ* (S1 Fig.) based on endogenous fluorescent pigments (pyoverdin, pyocyanin), representing an important marker for bacterial differentiation *in vivo* using FL imaging [48].

### PRODIGI quantitatively and longitudinally tracks changes in bacterial load in chronic wounds

**Fig 6:**
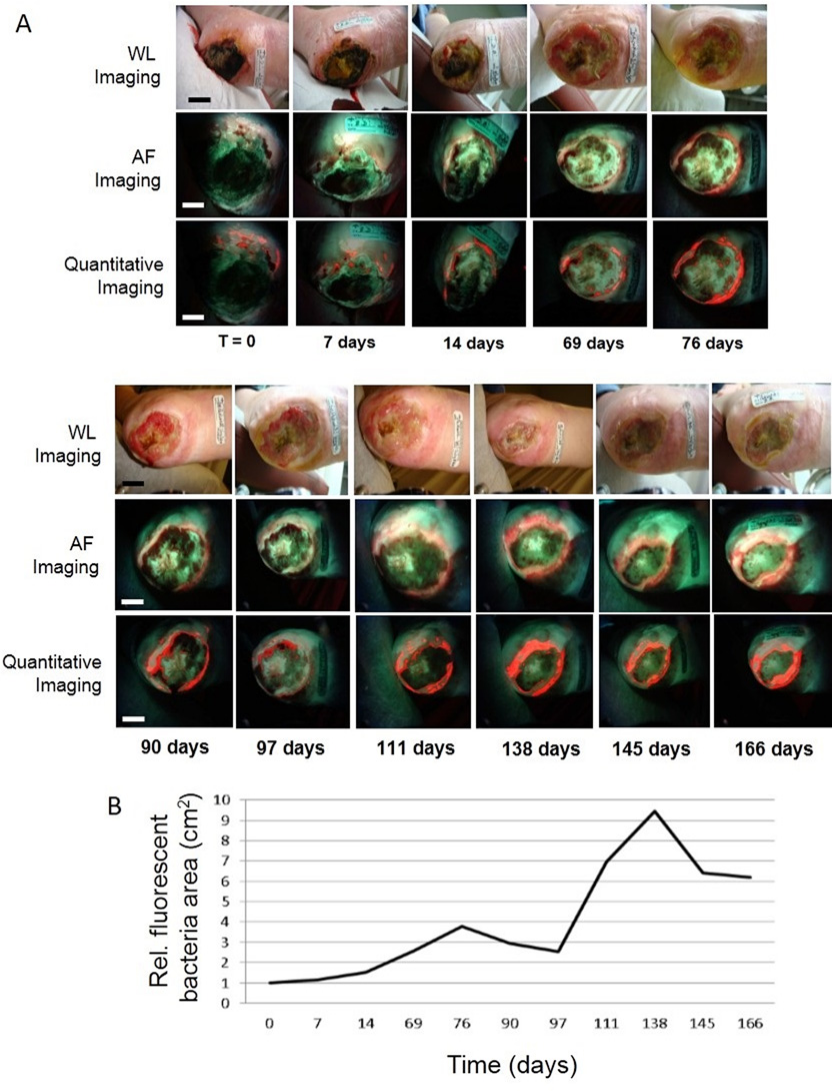
Quantitative longitudinal tracking of bacterial load in chronic wounds. *A*. Sequential white light (*top row*) and AF images (*middle row*) of a non-healing diabetic foot ulcer in a 67 y old female performed over 5.5 months. AF images revealed bluish-green fluorescence within the wound bed and bright red bacteria around the wound periphery. A fluorescence intensity-based segmentation algorithm was used to quantify bacterial load changes over time (*bottom row*), with the bacteria false-colored and overlaid on original AF images of the middle row. *B*. Quantitative changes in bacterial load over time measured by relative bacterial fluorescence amount (total red AF area in cm^2^) indicate clinically-significant microbial load in the wound periphery, both of which are missed during conventional clinical examination. *Scale bar*: *A*. ~*1 cm*.

In part 1, a non-healing wound was tracked in a 67 year-old female patient with a diabetic foot ulcer (Fig. 6). CSS examination determined that this wound was not infected and wound cultures were not ordered. Corresponding AF images revealed bluish-green FL within the wound bed and bright red FL in the wound periphery, confirmed for each time point by cultures to be moderate-to-heavy growth of P. aeruginosa and heavy growth of S. aureus, respectively. An intensity-based segmentation algorithm applied to the AF images quantified bacterial load changes over time. Quantitative changes in bacterial load over time, (total area in cm^2^) with red AF, indicated significant spatio-temporal changes in distribution and location of microbial load over half a year of frequent clinical visits, peaking at 138 days (Fig. 6B). These changes were not detected by WL examination and were not defined by changes in CSS.

**Fig 7:**
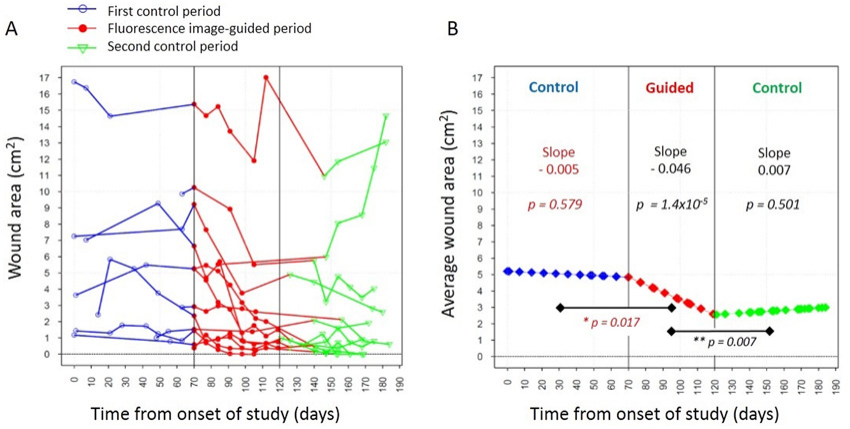
Fluorescence image-guided treatment accelerates wound closure over time compared with non-guided conventional treatment. *A*. Plot showing wound area measurements for twelve individual patients as a function of time from the onset of the study over the first control period (blue circles, the fluorescence image-guided period (red solid circles) and the second control period (green triangles). During the control periods, treatment was administered without real-time fluorescence image guidance. *B*. Plot showing the rate of change in the average wound area over the course of the study estimated from the regression model. The slopes and their corresponding *p-values* are shown for each study period. The data indicate that fluorescence image-guided treatment increased the rate of wound closure in a statistically significant manner compared with the control (non-guided) periods indicating that fluorescence image guided wound treatment could be beneficial addition to conventional wound therapy protocols. * *p-value* tests for change in the growth rate of average wound area in the guided period from the previous period. ** *p-value* tests for change in the growth rate of average wound area in the 2nd control period from the guided period.

We investigated whether FL imaging could be used to guide wound treatment (debridement with topical/systemic antibiotic) over time to reduce the bacterial load and help close wounds faster. In part 2, wound area was tracked in a separate group of 12 patients over approximately 6 months, comprising three contiguous 2 month periods, comparing treatment delivered without (control) and with FL guidance (guided). During the first standard treatment period (Fig. 7) there was a slight decrease in average wound area over time, but this was statistically insignificant (slope-0.005 cm^2^/day, p = 0.579). However, when FL imaging was used to target debridement and antibiotic application, a larger daily decrease in average wound area was found (slope-0.046 cm^2^/day), which was significantly different (p = 0.017) compared to the first standard control period (Fig. 7). When image guidance was again not used in the second control period we observed a slight daily increase in average wound area (slope +0.007 cm^2^/day), which was significantly different from the rate of change seen in the FL guided period (p = 0.007). For relative comparison, standard treatment had wound closure of 0.09 cm^2^ during the first period and a wound size increase of 0.21 cm^2^, respectively, over a 30 day period, whereas FL guided treatment had wound closure of 1.38 cm^2^ over the same period.

### Autofluorescence enables real-time quantitative guidance for wound cleaning and debridement

PRODIGI was used to obtain AF images before and after (S2 Fig.) wound cleaning procedures (e.g. saline irrigation and gauze scrubbing) to test the effectiveness in reducing bacterial load (S2 Fig.). We developed a prototype FL image analysis algorithm in MATLAB using texture and intensity-based segmentation [65] to quantitatively estimate total bacterial load (cm^2^) over time. This provided objective and quantitative image-guidance for wound cleaning (S2 Fig.) and debridement (S3 Fig.). Overall, real-time quantitative FL visualization of clinically-significant bacterial load (moderate-to-heavy growth) provided new knowledge about the temporal changes in density and localization of bacteria in the wound bed and periphery earlier than conventional methods.

## Discussion

High bacterial load is increasingly recognized as a major impediment to wound healing, further complicated by underlying impairments in host response mechanisms [6]. Bacteria also produce endotoxins, exotoxins, proteases, and local tissue injury that can prevent healing [49]. Better methods are needed for early diagnosis and management of chronic wound infections. Here, we report the first human studies using a handheld, portable FL imaging device for visualizing wound bacterial load in real-time, to reliably guide clinical decisions and procedures and to quantitatively monitor treatment response.

Notably, AF imaging detected clinically significant bacterial load in 85% of wound peripheries missed by conventional methods. Thus, the Levine method for swabbing only the wound bed may be insufficient, possibly resulting in antibacterial treatment being inappropriately withheld. However, modifying standard sampling practices to include swabbing of the wound periphery of all wounds would be impractical and costly. AF imaging could help clinicians decide if and where wound margins require sampling. PRODIGI also identified clinically significant bioburden in surrounding locations close to wounds, which represent sites of potential re-infection, where traditional methods do not examine or swab.

We further showed that CSS correctly identified clinically significant bacterial load in the wound bed 52.5% of the time compared to 74.6% for AF imaging (p = 0.003). CSS visually scans the wound periphery for tissue breakdown due to infection, and only swabs the wound bed. Our data demonstrate that while WL examination failed to detect signs of clinically significant bacterial load in the wound periphery or off-site 17.6% and 32.9% of the time, respectively, AF imaging accurately detected clinically significant bacterial load in 82.4% of wound peripheries and 67.1% of off-site areas (p < 0.001 for peripheries; p = 0.006 for off-site). Undetected, such bioburden may prevent wound healing due to persistent protease activity in the wound edge [50] and risk re-contamination of a treated wound. Our data suggest a limited value of obtaining a bacterial count at a single location as per best practice methods, which corroborates other reports [51].

WL examination missed ~50% of wounds with clinically-significant levels of bacteria that were detected by AF imaging. AF imaging may be sensitive to bacterial load that has not yet manifested into perceptible CSS under WL [52]. Overall, while WL examination was good at judging the presence of clinically significant bioburden within the wound bed (PPV 94.5%), sampling of the wound bed for microbiology was indicated only 14% of the time (sensitivity), while AF indicated testing in 100% of cases, of which 74.5% were true positive as confirmed by microbiology (p < 0.001). Thus, the higher predictive value of standard methods alone comes at the cost of missing many swabs that have clinically significant bacteria levels. Moreover, longitudinal FL data showed that 90% of patients would have been sent home prematurely with a clinically significant bacterial load at least once during the care cycle if assessed by CSS, potentiating a missed opportunity to treat the wound earlier.

Identifying and quantitating wound bacterial burden is an important determinant of infection and healing [1]. Our data on the visualization and quantitative tracking of bacterial load led to the identification of a novel, simple method for image-guided debridement and topical application of antibiotic and antiseptic, which minimizes unnecessary trauma to the wound boundary and maximizes the contribution of debridement to reducing bacterial burden [1,53,54]. Every wound has the potential for infection, but distinguishing true infection from critical colonization by best practice methods remains challenging and arbitrary, and can lead to over- and under-treatment [55–57]. Multiple variables including host response, local and systemic factors, malperfusion, immunosuppression, diabetes, and medications affect the risk of infection. Critically colonized wounds can be difficult to diagnose because they do not always display classical signs of infection or clearly elevated levels of bioburden [55–57]. Indeed, the clinical relevance of differentiating critically colonized wounds from infected wounds remains controversial [11,56]. Identifying a high bacterial load in asymptomatic patients before infection occurs using AF imaging may help prevent infections by prompting aggressive treatment. If a bacterial infection is suspected, antibiotic selection could be guided by the established clinical principles [58] and by AF identification of heavy bacterial burden and differentiation between Gram negative P. aeruginosa and Gram positive S. aureus.

The present study used nascent technology in a relatively small number of patients, representative of the general population of patients with chronic wounds, to test safety and feasibility and investigate the first clinical use of AF imaging for wound assessment. Hence, the sample size of 28 patients was not based on *a priori* considerations of statistical power, but does meet the minimum number of subjects suggested by Browne to estimate parameters of interest in a pilot study (inclusion/exclusion criteria, recruitment, randomization, treatment, compliance, follow-up and selection of ideal primary outcome) for the design of larger studies [59]. It also provides the rationale for further development of quantitative FL technology.

AF imaging for clinical wound assessment does have limitations. Firstly, at 405 nm excitation, FL is only detected to a depth of ~1–1.5 mm. Detecting deeper bacterial load non-invasively is challenging, unless used for FL-guided debridement. Secondly, while PRODIGI can detect and differentiate light, moderate and heavy bacterial growth from occasional growth, currently it cannot quantitatively indicate clinically significant bacterial concentration in the wound (e.g. 10^6^ CFU/gm of tissue). In the future, a disposable FL adhesive ‘sticker’ placed close to the wound will be used to calibrate bacterial AF signals, to define an optimal cutoff value for missing high bacterial levels and over-calling commensal levels. The technology can also be adapted for exogenous contrast agent-based assays. For example, the use of the prodrug aminolevulinic acid (ALA), which causes endogenous biosynthesis of the fluorophore protoporphyrin IX in many bacterial species [60,61] may provide a clinically-compatible method to increase the detection sensitivity of low levels of microbes.

Thirdly, PRODIGI’s ability to identify specific bacterial species is limited by the use of endogenous AF signals, preventing differentiation between commensal and pathogenic bacteria, due to a shared presence of endogenous fluorophores across species. However, as previously mentioned, microorganisms can lose and acquire pathogenic (virulence) genes [62] and commensal microbes can become pathogenic in the right setting [63]. It has yet to be determined whether identifying the diversity of microorganisms in a wound significantly impacts diagnosis and treatment. Finally, PRODIGI images are displayed in red and green. While not experienced in this study, color blind individuals cannot read the images. To solve this, software can be implemented to generate grey-scale output.

In conclusion, this is the first clinical use of AF imaging for chronic wound assessment, treatment guidance and response monitoring. Our data suggest that standard procedures for detecting infection are suboptimal. As a novel *point-of-care* imaging device for real-time diagnosis, assessment and management of wound infections, PRODIGI could improve the standardization of current wound assessment and treatment guidance. It identifies pathogenic microbes, differentiates between two major pathogenic species (P. aeruginosa and S. aureus*)*, and informs medical treatment decisions. PRODIGI could also offer a quantitative and reproducible way to monitor the effectiveness of existing and emerging wound care treatments.

Our early data indicate FL image-guided wound treatments can accelerate wound closure, although the underlying biological mechanisms require further investigation in larger patient samples. Moreover, PRODIGI may offer a new wound care research tool to objectively evaluate emerging antimicrobial wound treatments. Its role in the early detection and prevention of complications from nosocomial infections still needs to be explored [64]. Overall, these results demonstrate that PRODIGI is a simple, effective technology that can overcome previous technical and clinical limitations of wound infection assessment and shows promise for more accurate FL image-guided wound sampling, cleaning and debridement, and monitoring of treatment response.

## Supporting Information

S1 Fig Autofluorescence imaging differentiating P. aeruginosa from other species. *A*. The PRODIGI white light image shows chronic wounds on a non-diabetic 91 y old male patient’s right ankle. *B* corresponding AF image distinguished the endogenous red fluorescent porphyrins in S. aureus (smaller circle) from the green fluorescent pigments in P. aeruginosa (larger circle). *C*. Corresponding point fluorescence spectra confirming that while both species emit green fluorescence between 490–550 nm with 405 nm excitation, P. aeruginosa emits a distinct bright bluish-green fluorescence peaking at 480 nm unlike S. aureus, which emits a distinct porphyrin red fluorescence > 600 nm. *Scale bars*: *A*,*B*. *1 cm*.
(JPG)

S2 Fig Autofluorescence image guidance of wound cleaning. *A*. White light image of a chronic wound on the left breast of 21 y old female with pyoderma gangrenosum showing no visible bacteria. *B*. Corresponding AF image showing the location of bacterial load (heavy growth *S*. *aureus*) used to guide cleaning with saline and gauze. *C*. Immediately after cleaning, AF imaging shows persistent bacteria beneath the skin surface (1–2 mm depth) even, possibly indicating the need for additional debridement. *D*,*E*. Quantitative fluorescence images of the fluorescent bacterial area before and after cleaning. *Scale bar*: *A-E*. *1 cm*.
(JPG)

S3 Fig Autofluorescence image guidance for surgical wound debridement. *A*. Photograph of standard debridement procedure of a diabetic foot ulcer in a 62 y old male patient. *B*,*C*. White light corresponding AF images and quantitative AF images of bacterial load before and after debridement over two clinical visits, 3 weeks apart. Heavy S. aureus bioburden is seen in the wound periphery in the AF images both pre- and post-debridement, confirmed by microbiology. Bacterial fluorescence increases deep into devitalized tissues at the wound periphery even after debridement, undetected by white light visualization. Bacterial load decreases markedly in the wound periphery ~20 days later. *D*. Quantitative changes in bacterial load can be obtained before, between and after wound debridement procedures. *Scale bars*: *B*,*C*. *2 cm*.
(JPG)

S1 Protocol UHN/JDRTC: RESEARCH PROTOCOL. (PDF)

S1 STARD Checklist STARD checklist for reporting of studies of diagnostic accuracy. The STARD checklist describes the design of the current study in order to improve reporting accuracy and completeness. The associated flow diagrams are presented in Figs. 1 and 2.
(DOC)
