# Supplementary figures and images for: Point-of-Care Autofluorescence Imaging for Real-Time Sampling and Treatment Guidance of Bioburden in Chronic Wounds: First-in-Human Results
Source: PLoS One. 2015 Mar 19;10(3):e0116623. doi: 10.1371/journal.pone.0116623 (PMC4366392; doi:10.1371/journal.pone.0116623)

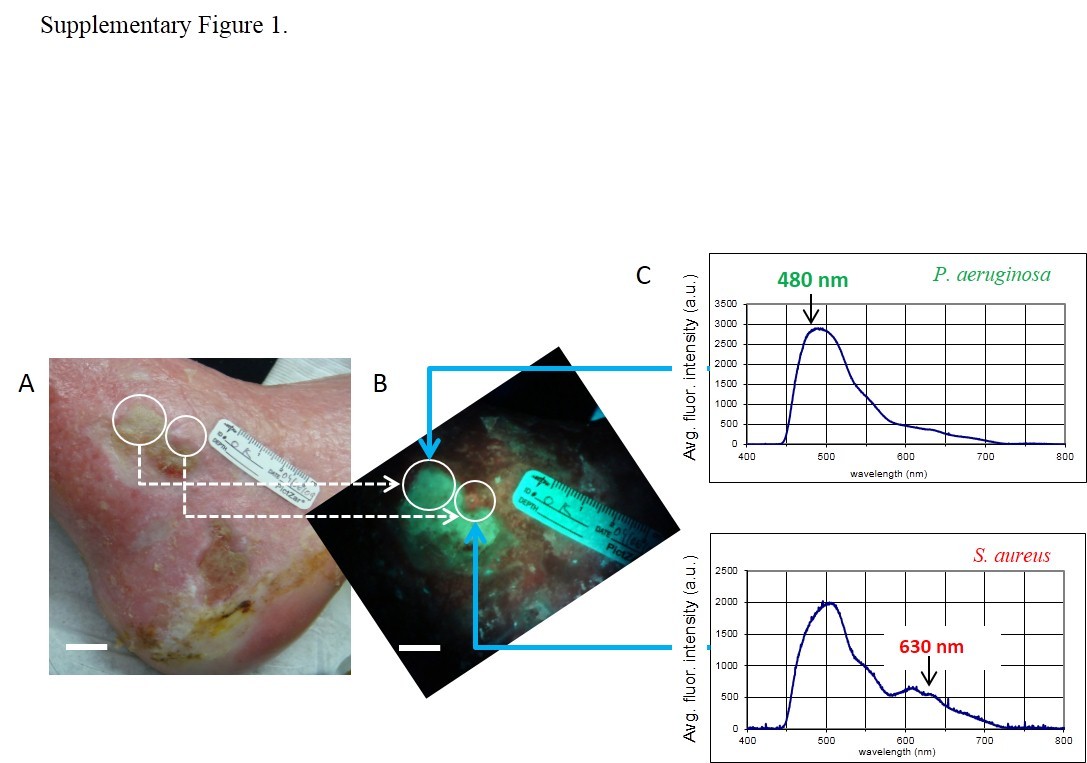

Supplement: S1 Fig — A. The PRODIGI white light image shows chronic wounds on a non-diabetic 91 y old male patient’s right ankle. B corresponding AF image distinguished the endogenous red fluorescent porphyrins in S. aureus (smaller circle) from the green fluorescent pigments in P. aeruginosa (larger circle). C. Corresponding point fluorescence spectra confirming that while both species emit green fluorescence between 490–550 nm with 405 nm excitation, P. aeruginosa emits a distinct bright bluish-green fluorescence peaking at 480 nm unlike S. aureus, which emits a distinct porphyrin red fluorescence > 600 nm. Scale bars: A,B. 1 cm. (JPG) [file pone.0116623.s001.jpg]

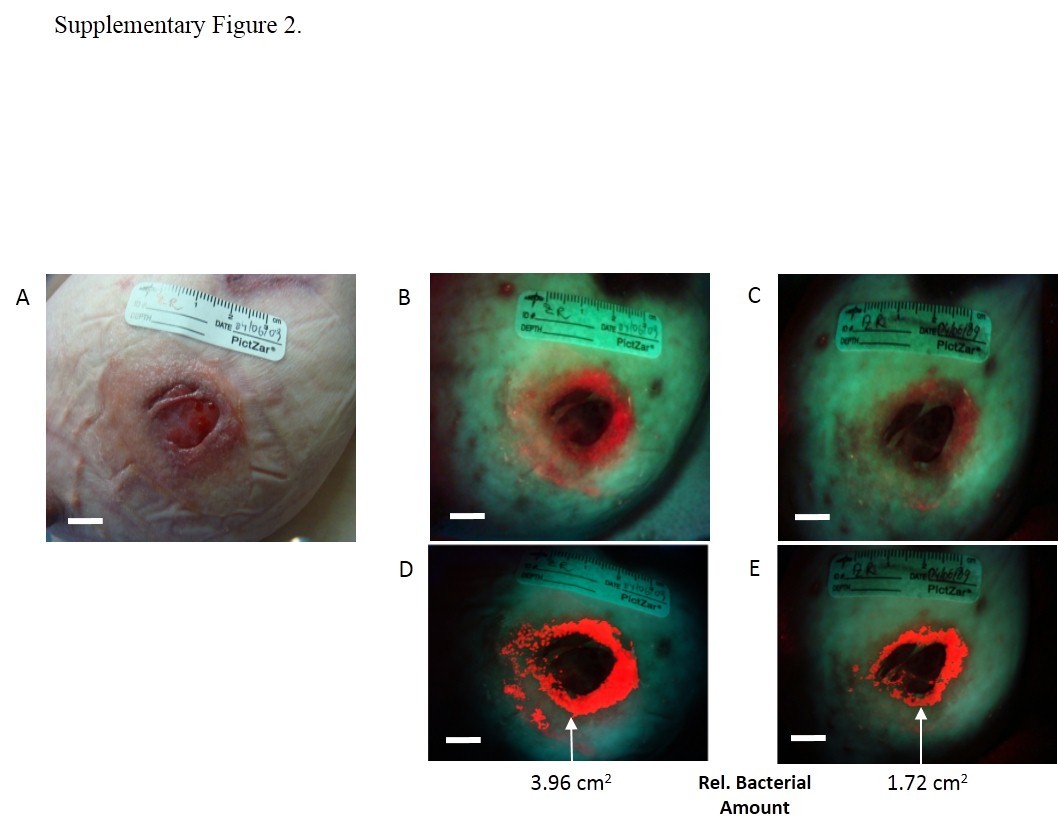

Supplement: S2 Fig — A. White light image of a chronic wound on the left breast of 21 y old female with pyoderma gangrenosum showing no visible bacteria. B. Corresponding AF image showing the location of bacterial load (heavy growth S. aureus) used to guide cleaning with saline and gauze. C. Immediately after cleaning, AF imaging shows persistent bacteria beneath the skin surface (1–2 mm depth) even, possibly indicating the need for additional debridement. D,E. Quantitative fluorescence images of the fluorescent bacterial area before and after cleaning. Scale bar: A-E. 1 cm. (JPG) [file pone.0116623.s002.jpg]

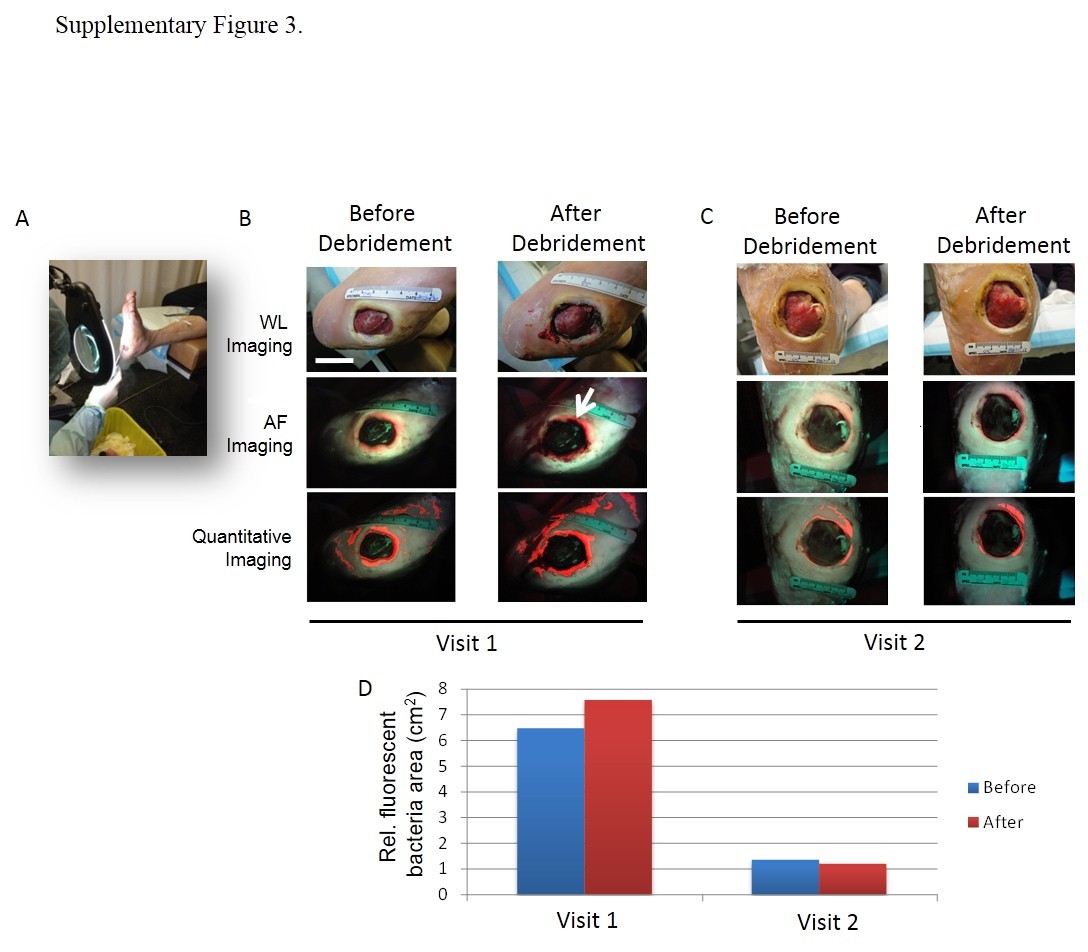

Supplement: S3 Fig — A. Photograph of standard debridement procedure of a diabetic foot ulcer in a 62 y old male patient. B,C. White light corresponding AF images and quantitative AF images of bacterial load before and after debridement over two clinical visits, 3 weeks apart. Heavy S. aureus bioburden is seen in the wound periphery in the AF images both pre- and post-debridement, confirmed by microbiology. Bacterial fluorescence increases deep into devitalized tissues at the wound periphery even after debridement, undetected by white light visualization. Bacterial load decreases markedly in the wound periphery ~20 days later. D. Quantitative changes in bacterial load can be obtained before, between and after wound debridement procedures. Scale bars: B,C. 2 cm. (JPG) [file pone.0116623.s003.jpg]
