# Supplementary material for: Point-of-Care Autofluorescence Imaging for Real-Time Sampling and Treatment Guidance of Bioburden in Chronic Wounds: First-in-Human Results
Source: PLoS One. 2015 Mar 19;10(3):e0116623. doi: 10.1371/journal.pone.0116623 (PMC4366392; doi:10.1371/journal.pone.0116623)
Supplement: S1 Protocol — (PDF) [file pone.0116623.s004.pdf]

UHN/JDRTC: RESEARCH PROTOCOL

Evaluation of a 'hand-held' fluorescence digital imaging device for real-time advanced wound care monitoring.

Investigators: Dr Ralph S. DaCosta[1] (PI), Dr Ron Linden[2], Dr Cheryl Rosen[3], Dr Ludwik Fedorko[2]

[1] Div. of Biophysics and Bioimaging, Ontario Cancer Institute, University Health Network, 610 University Avenue, Toronto, Ont. M5G 2M9,
Tel. (416) 581-8645, Fax. (416) 946-6529.
Emails: rdacosta@uhnres.utoronto.ca

[2] The Judy Dan Research & Treatment Centre, 555 Finch Avenue West, 2nd Floor, (former Branson Hospital), Toronto, Ontario, M2R 1N5
Tel. 416 223-6600, Fax. 416 223-6764
Email: hbot@bellnet.ca and ronlinden@mac.com (for RL) and Ludwik.Fedorko@uhn.on.ca (for LF)

[3] Dermatology, Toronto Western Hospital, University Health Network, Toronto Western Hospital, East Wing, 8th Floor Rm. 517, 399 Bathurst St.Toronto, Ontario, Canada M5T 2S8.
Tel. (416) 603-5800 x5952, Fax. (416) 603-2782.
Email: cheryl.rosen@uhn.on.ca

Table of Contents

1. Introduction and Rationale 3
2. Study Objectives and Specific Aims 7
  Specific Aims: 7
3. Overall Study Design 8
  Hypothesis 8
  Clinical Setting and Patient Enrollment Criteria 8
    Diabetic Wounds: 9
  Specific Study Procedures 10
    Standard White Light-Based Clinical Wound Assessment 10
    Fluorescence imaging and spectroscopy measurements 12
    Swabbing and Bacteriology 15
    Question Based Survey 16
    Handling of Research Equipment During Study 17
    Longitudinal Imaging of Each Patient 17
    Study Variables, End Points and Outcomes 19
    Data Collection 21
    Intervention 21
    Adverse events 22
4. Data Storage and Statistical Analysis 23
5. Potential Impact 25
6. Bibliography 27

1. Introduction and Rationale

Wound care is a major clinical challenge and presents an enormous burden to health care worldwide[1-3]. As wounds (chronic and acute) heal, a number of key biological changes occur at the wound site at the tissue and cellular level [2]. Among these are inflammation, reformation of the epidermal barrier, and remodeling of the connective tissue in the dermis. However, a common major complication arising during the wound healing process, which can range from days to months, is bacterial infection [2, 3]. This can result in a serious impediment to the healing process and lead to significant complications, especially in chronic non-healing wounds. Currently, the standard wound care includes monitoring for possible infection by direct visual inspection under white light and by taking samples for analysis in the laboratory which takes approximately two days to provide a result. However, qualitative visual assessment only provides a gross view of the wound site (*i.e.*, presence of purulent material and crusting) but does not provide the critically important information about underlying changes that are occurring at the tissue and cellular level (*i.e.*, infection, matrix remodeling, inflammation, and necrosis).

All chronic wounds contain bacteria, but identifying whether the wound is in bacterial balance (e.g. contamination with organisms on the surface or colonization with organisms in the tissue arranged in micro colonies without causing damage) or bacterial imbalance (e.g. critical colonization and infection) is of primary clinical importance as it is a major determinant of the rate of healing. Also, it is important to note that there is a continuum of bacterial presence progressing from bacterial balance to imbalance leading to bacteria-mediated tissue damage in a chronic wound. These bacteria include common species typically found at wound sites (*i.e.*, Staphylococcus and Pseudomonas species) [1, 2, 10, 11]. The diagnosis of infection is typically made clinically, based on standard signs and symptoms [3] identified during examination in and around the local wound bed, the deeper structures, and the surrounding skin by the wound care team. However, a major problem in conventional practice is that bacteria within and around a

wound cannot be directly visualized with the unaided eye under standrad room lighting with white light. Diagnosis is typically informed by clinical signs and symptoms of infection based on the symptoms caused by bacterial contamination and/or infection within the wound (*i.e.*, pain, purulent exudate, crusting, swelling, erythema, heat) [2-4]. On suspicion of infection, the wound care team will often sample the wound using either surface swabbing or biopsy for clinical microbiological testing.

Bacterial swabs are collected at the time of wound examination and have the advantage of providing identification of specific bacterial/microbial species and quantification of bacterial burden. However, often, multiple swabs are collected randomly from the wound site and these are not targeted, and some swabbing techniques may spread the microorganisms around with the wound during the collection process, thus affecting patient healing time and morbidity. This may be a problem especially with large chronic (non-healing) wounds where the detection yield for bacterial presence is suboptimal, despite the collection of multiple swabs [15]. Furthermore, bacteriological culture results often take about 2-4 days to come back from the laboratory, thus significantly delaying diagnosis and treatment [15]. Thus, bacterial swabs do not provide real-time detection of infectious status of wounds. In addition, although wound swabbing appears to be straightforward, it can lead to inappropriate treatment, patient morbidity and increased hospital stays if not performed correctly. Therefore, an image-based method that allows real-time monitoring of wound healing, particularly early dermal connective tissue remodeling, and the presence of bacterial contamination and/or infection over time could have a significant clinical impact.

Another aspect of bacterial impact on wound healing relates to connective tissue breakdown. The remodeling and healing of connective tissues in wounds involves simultaneous synthesis and degradation of collagen fibrils [8, 9]. This degradation process is driven by bacterial imbalance with the host which leads to increased enzymatic-breakdown by proteases released by the bacteria. Changes in wound connective tissue (e.g. collagen in the surrounding

skin about a wound) can be useful in helping to determine the extent of tissue breakdown caused by bacteria. However, currently there is no means of accessing connective tissue composition (or integrity) available to wound care personnel. Thus, an imaging device which could determine healthy from degraded connective tissue within and around a wound would be useful for diagnosis.

Autofluorescence imaging provides a powerful means of visualizing both bacteria and connective tissue in real-time. Autofluorescence has been used previously (by our own team) in other clinical applications, for example, in gastroenterology to image both collagen and bacterial fluorescence in clinical studies [5-7]. Thus, we wish to expand the use of tissue autofuorescence imaging technology to wound care and management in order to obtain biologically relevant information about the wound site at the tissue and biomolecular levels in real-time during the healing process [5-7]. When used to assess wounds, tissue autofluorescence may aid in determining the degree of wound healing (e.g. by visualizing and measuring connective tissues) and the presence of bacterial infection.

In preliminary preclinical testing, we have discovered that when wounds are illuminated by violet/blue light, endogenous collagen in the connective tissue matrix emit a characteristic green fluorescent signal [6], while most pathogenic bacterial species emit a unique red fluorescence signal due to the production of endogenous porphyrins [5]. Therefore, with autofluorescence imaging, no exogenous contrast agents are needed during imaging, making this approach particularly appealing as a diagnostic imaging method for clinical use.

We have recently developed an innovative optical molecular imaging platform (called PRODIGI™) based on high-resolution fluorescence and white-light technologies in a hand-held, real-time, high-resolution, non-invasive format. PRODIGI™ offers a non-contact means of obtaining instantaneous image-based measurements of diagnostically-relevant biological and molecular information of a wound and surrounding skin tissues for the first time and could have significant impact on improving conventional wound care, management, and guidance of

intervention. Based on extensive preclinical studies in our labs, PRODIGI™ has demonstrated its capability at collecting autofluorescence images of wounds and detecting the presence and relative changes in connective tissue (e.g. collagen) content and bio-distribution involved in wound healing. It can also detect the presence and relative amounts of commensal and pathogenic bacteria within the wound based on autofluorescence alone (these bacteria are invisible to standard visualization with the naked–eye using white light), thus providing a measure of infection status. This could significantly impact clinical wound care and management by i) reducing the complications associated with missed detection of bacterial infection under conventional practice, ii) facilitating image-guided wound sampling by targeted swabbing/biopsy and iii) monitoring wound healing and treatment response over time.

Previous Related Studies: A pilot-level clinical study (UHN REB protocol # 09-0015-A, PI: DaCosta, 50 patients imaged to date) performed by our group from 2008-2011 to assess the clinical utility of the device successfully demonstrated that tissue autofluorescence produced by endogenous collagen/elastin in the skin appears green in the fluorescent images, while most clinically-relevant bacterial colonies present in the wound produce a red fluorescence signal [6] caused by endogenous porphyrins. Some bacterial species (e.g. *Pseudomonus aerogenosa*) produce a green fluorescence signal that can be differentiated spectrally and texturally from the fluorescence of the dermis (another hue of green, discernable by our proprietary image analysis software). The PRODIGI™ device is sensitive enough to detect these green and red fluorescence signals from tissue and bacteria confirming the utility of this compact and portable imaging platform for clinical wound care.

An Investigational Testing Application (ITA) has been submitted to Health Canada for the use of PRODIGI™ at the Judy Dan Research and Treatment Centre (JDRTC), in accordance with the protocol described below. A copy of the No-Objection Letter will be forwarded to UHN REB and JREB when it becomes available. The No-Objection Letter is expected to be granted by 30 May 2012.

2. Study Objectives and Specific Aims

The primary objective of this clinical study is to evaluate the use and effectiveness of the UHN handheld PRODIGI™ imaging device for real-time and non-invasive detection and tracking of pathogenic bacterial presence, contamination and infectious status in complex wounds over time. This will enable us to determine if PRODIGI™ can detect and longitudinally and quantitatively track intrinsic changes that may occur during the wound healing process including, but not limited to, collagen re-modeling and bacterial infection of the wound site.

As a secondary objective, based on findings to date from past clinical studies (UHN REB 09-0015-A), we now wish to determine if PRODIGI™ can detect the presence of clinically-significant (critical colonization or infection) using fluorescence and then that information may be used by the clinical team to effectively deliver a clinical intervention to treat the infection.

As a third objective, we wish to obtain valuable end-user data on the clinical utility of the device within the wound clinic environment with a brief question-based survey. This information will be used to optimize subsequent versions of PRODIGI™ during product development and to improve the integration of the technology from the perspective of both the clinician and the patient.

Most importantly, our aim is to quantitatively verify the added value that this imaging device brings to traditional wound care practice and to what extent it will change the gold standard (microbiological swabbing) of clinical wound diagnosis.

Specific Aims:

1) To determine if the fluorescence imaging device can detect changes in connective tissue (e.g. collagen) over time that can be correlated with wound healing or re-modelling,

compared with changes in wound size over time.

2) To determine the effectiveness of the fluorescence imaging device in detecting the presence (or contamination) of bacteria in and around a wound (including infection), compared with standard best practice methods using white light visualization and clinical signs and symptoms, with swabbing and bacteriology as the 'gold standard'.

3) To identify the relationships between autofluorescence imaging of collagen and bacteria in wounds (including the wound margin) and the following: i) clinical signs of infection, ii) microbial load (i.e., number of organisms per gram of wound tissue), and iii) diversity of microbial species in the wound (i.e., number of different species isolated per wound), and Gram signing.

4) To assess the potential utility of the device to guide intervention and alter the course of treatment in wounds that are infected.

3. Overall Study Design

Hypothesis

We hypothesize that real-time imaging of tissue autofluorescence signals emanating from endogenous connective tissue (e.g. collagen) and pathogenic bacteria within complex wounds can be used to determine healing status (*i.e.*, collagen re-modeling and wound closure), detect wound bacterial contamination and/or infection that is occult under standard clinical white light evaluation, and guide intervention during wound care.

Clinical Setting and Patient Enrollment Criteria

Diabetic Wounds:

Patients will be examined at The Judy Dan Research & Treatment Center (JDRTC) (formerly named The Judy Dan Wound Care Clinic) at 555 Finch Avenue West, Toronto) under the direct supervision of the investigators (DaCosta, Wilson, and Linden). All patients who will be treated by staff physicians at JDRTC will be considered for eligibility into study. Eighty new patients with wounds will be entered into this trial, based on statistical power-based calculations for sample size determined in collaboration with the UHN Biostatistics Group (assuming one wound per patient). Recruitment of patients will continue throughout the study duration, until the total number of patients has been met. Recruitment of patients will be directed by Drs. Linden and Fedorko. After the recruitment process, consent will be obtained by a member of the UHN research team. The consent process will be documented in the Informed Consent Form Checklist as per UHN Best Clinical Practice Guidelines.

Patients will be included in the study according to the following criteria:

- > 18 years of age
- males and females
- new patient to the JDRTC to ensure consistent work-up procedures (as described below) prior to treatment
- presenting with acute or chronic wounds (i.e., diabetic ulcers or other), with known or unknown infection status.

Patients will be excluded in the study according to the following criteria:

- treatment with an investigational drug within 1 month before study enrolment
- any contra-indication to routine wound care and/or monitoring
- inability to consent

A translator will be provided to patients who are unable or uncomfortable communicating in English.

Specific Study Procedures

Standard White Light-Based Clinical Wound Assessment

Patients who meet the entry criteria (determined by staff at the JDRTC) with chronic wound problems will be informed and consent will be obtained to participate in this trial by the UHN research staff (Consent Form and Study Instrument Document are attached). In our experience, the majority of patients at the JDRTC have presented with diabetic-related chronic wounds in the lower extremities and so we expect to enroll a majority of the study patients with foot ulcers. Such patients are typically enrolled for treatment at the JDRTC and wait about 2 – 3 weeks prior to receiving treatment (herein called intervention) such as hyperbaric oxygen treatment, antibiotics, debridement, ultrasound therapy, etc. Throughout their enrollment at the JDRTC, patients will have their wounds prepared by standard methods, and will be assessed for standard clinical signs and symptoms (CSS) of localized infection in their wounds by the attending clinician or trained wound care specialist, according to standard clinical practice employed at the treatment site. Clinical staff will take the patient's medical history, including age and gender. Demographic data (e.g. diabetes) will be collected from the patient record (patient identification will be maintained using a unique subject/patient identification number). In addition, wound variables known to be indicators of wound healing and wound infection will be measured including type of chronic wound, wound size, and amount of necrotic tissue. Chronic wound type will be categorized based on etiology and will include pressure ulcers, venous ulcers, diabetic ulcers, chronic secondary incisions, or chronic traumatic wounds. Wound

etiology will be determined using clinical history, physical exam, and available diagnostic exams (e.g., ABI, PVR, Doppler studies, angiography, or x-rays), if required.

Data regarding the type, location, and history of the study wound will be collected from direct observation, the patient record and patient/caregiver report by the clinical team. In order to measure wound depth, a cotton-tipped swab will be placed in the deepest area of the wound and marked at the point level with the surrounding peri-wound skin. For chronic diabetic wounds the type and amount of wound bed tissue will be measured using the necrotic tissue subscale of the Pressure Sore Status Tool (PSST) [12]. Necrotic tissue will be defined as yellow, tan, brown, gray or black tissue in the wound bed after cleansing the surface of the wound bed with saline-moistened gauze, which ensured the necrotic tissue will be adherent [13]. The category descriptors on the 5-point scale were: 1 = none present; 2 = <25-percent wound bed covered; 3 = 25- to 50-percent wound bed covered; 4 = >50 and <75-percent wound bed covered; and 5 = 75- to 100-percent wound bed covered.

Surface area and depth will serve as measures of wound size. Surface area will be measured by tracing the wound edge on the white light image of the wound (and labeled with patient ID, date and wound ID) using an indelible marker with a 1 mm tip. The wound edge will be defined as the line dividing the healed portion from the unhealed portion of the wound. The depth of the wound will be measured using a cotton-tipped swab placed in the deepest portion of the wound. The amount of necrotic tissue in the wound bed will be rated using direct observation, and the PSST. The presence or absence of the clinical signs and symptoms of infection will be assessed using the Clinical Signs and Symptoms Checklist (CSSC) [14] and each wound will be categorized as infected or non-infected by the researcher based on the clinical interpretation of this assessment. When deemed appropriate by the clinical staff the Bates-Jensen Wound Assessment Tool (BWAT) score may also be evaluated by the clinicians and recorded by the research staff in the Care Report Form in order to aid quantification of wound severity and its change over time. BWAT scores will be used a comparative tool with

wound infection diagnosis made with PRODIGI™. For example, low BWAT scores are expected not to be infected, and we will establish if this is true based on the fluorescence imaging and microbiology test results for each wound of a patient during multiple visits (if BWAT scores are available).

For subjects with more than one eligible chronic wound (*i.e.*, full-thickness and non-arterial), one wound will be randomly selected *(i.e.*, random draw) for data collection procedures. If other 'eligible wounds' are available for a single patient, imaging of this wound will be conducted at the discretion of the physician and based on the time available to the clinical team for the additional procedure. All study data will be collected by a member of the research team and will be recorded on a Case Report Form labeled with subject/patient identification number only, which will be kept in secure storage (double locked) location during the study at the JDRTC.

After the initial data is collected for each wound, a high-resolution white light photograph (using a length scale placed in the field of view to aid in wound size measurements) will be taken of each wound using the imaging device and printed on a color printer, immediately after the wound has been examined. The clinician (or wound care personnel) will be asked to draw (using an indelible ink pen) on the photograph to indicate the area he/she considers infected with bacteria and thus would normally take a bacterial swab or biopsy (each photo will be labeled with patient ID, date and wound ID). However, no swabs or biopsies will be taken at this time. Immediately after the physician has 'committed' to their decision of bacterial infection on the wound and areas that would require swabbing for microbiology confirmation at diagnosis, PRODIGI™ will subsequently be used to take fluorescence images of the wounds.

Fluorescence imaging and spectroscopy measurements

We will perform fluorescence imaging of selected wound sites in each patient following standard white light-based clinical assessment using the prototype device. Fluorescence imaging with the device and point-spectroscopy measurements will be performed on the selected wound from a patient's limb as well as a non-wounded contralateral limb (as a control) from a single patient.

The PRODIGI™ device used here allows non-contact and non-invasive imaging of the wound surface, and images are collected in approximately 1-2 seconds using both white light (from a standard camera flash) and two violet-blue (405 nm +/- 40 nm emission, narrow emission spectrum) LED light arrays to illuminate the tissue from a distance of about 10 cm, thus causing the tissue constituents to emit a fluorescence signal. The total optical power density on the skin surface is approximately 0.005 W/cm^2, based on an output power of approximately 2 Watts each, emanating from circular LED with a radius of 0.5 cm. There is no known potential harm to either the wound (or skin surface) or the eyes from the blue (405 nm) wavelength light or the power density of this light used in this device. However, the users of the device will ensure that it is never pointed directly at any individual's (patient /staff) eyes during imaging procedures (Refer to Fig. 1 in Study Instruments Document). It should also be noted that 405 nm light does not pose a risk to health according to international standards formulated by the International Electrotechnical Commission (IEC) [17], who specify that exposure of up to 0.2 W/cm^2 is safe for skin. Following examination of the wound by fluorescence imaging using the device, a color photograph of the wound will be printed and all wound areas containing ANY red fluorescence (i.e. bacteria present) [6] will be marked by the research staff using an indelible ink pen. These areas may include wound areas not previously marked by the clinician on the white light photos of the same wound, and this would indicate that fluorescence imaging identified bacteria in areas not considered infected under standard white light infection.

As demonstrated by our previous study (UHN REB protocol 09-0015-A) the PRODIGI™ device is sensitive enough to detect these green and red fluorescence signals and records the

images into its digital memory card for analysis afterwards. If bacteria are detected on the second pass (using the fluorescence imaging device and spectroscopy measurements where appropriate) which are undetected during the standard clinical wound assessment procedure (first pass), then bacterial swabbing and/or tissue biopsy will be obtained from these bacteria-red and bacteria-green fluorescence positive areas and sent for bacteriology testing to a blinded microbiology lab. Conversely, if bacteria are not detected on the second pass using fluorescence imaging but were detected during the standard clinical wound assessment procedure (first pass), then bacterial swabbing and/or tissue biopsy will be obtained from these areas and also sent for bacteriology testing to a blinded microbiology lab.

Multiple point fluorescence spectroscopy measurements will be made, at the discretion of the research team, to determine specific autofluorescence spectral signatures of unique fluorescence features observed during imaging. This will be within and around the wound using a custom-built laptop computer-controlled portable ‘spectroscopy’ device (*See Fig. 2, Study Instruments*). If deemed appropriate, areas of the wound bed and edge will be measured using the spectroscopy probe. Fluorescence imaging and spectroscopy measurements require that the room lights be dimmed or turned off during the examination for a brief time, to minimize the background light.

This device uses an optical fiber probe, which is held close to the wound surface (but not in contact) to collect the fluorescence signal from the wound and sends this information to the spectrometer which separates the light signal into different colors (wavelengths), and this data is stored on the laptop computer. Tissue spectra will be collected using the same blue light LED (405 nm emission) of the fluorescence imaging device. This measurement will be made after the fluorescence digital imaging is completed for each wound, and is estimated to take about 1-2 minutes. Spectra will be collected from areas of interest as determined by the fluorescence imaging device. Any areas that appear abnormal under fluorescence (e.g. suspected application of a topical skin cream, lotion, etc.) will also be measured to create a database of

possible confounding non-biological autofluorescence signals. Each area measured by spectroscopy will be documented on a digital white light image of the wound area for comparison with bacteriology results. The fluorescence spectroscopy data will yield quantitative spectral information as to the relative intensity and contribution of the green and red fluorescence to the total fluorescence signal measured at the wound. The fluorescence imaging and spectroscopy data obtained from these wound measurements will be stored on an encrypted laptop computer for subsequent analysis, which will be stored in a secure (double-locked) location at the JDRTC.

Swabbing and Bacteriology

A swab or biopsy will be collected from each area marked as being suspicious for infection in the printed color photos of both the white light and autofluorescence images taken with the prototype device. The swab/biopsy will be done in an area which has consensus from at least two study staff in order to determine the bacterial burden in each marked area. The study staff will indicate consensus by both initialing the fluorescence image after the areas have been marked.

For each marked area (e.g. from the white light and fluorescence color photos), a bacterial swab or a specimen of viable wound tissue will be removed aseptically using a standard sterile swab or a dermal punch instrument, and this will be determined by the wound care specialist/physician. The tissue specimen will be placed in sterile container and transported to the microbiology laboratory for processing by the end of the day. These will be used for quantitative bacterial culture analyses within the wound bed and from surrounding normal tissue. Wound cultures will be processed at a single microbiology laboratory associated with the JDRTC. Wound cultures (swabbing/biopsy) will be used to identify bacterial species present in and around the wound, as well as the relative number of colony forming units (CFU) and the

gram signing of the bacteria. All organisms isolated will be identified using standard microbiologic procedures, which are based on criteria such as colony morphology and gram stain appearance [15].

Therefore, for each patient in this study, a complete data file will include (for each visit to the clinic): white light visualization and clinical signs and symptoms of each wound examined (performed by the clinician), white light and corresponding autofluorescence images of the wound obtained using the prototype device, corresponding fluorescence spectra (performed by research staff when appropriate) and bacteriology results obtained from swabbing/biopsy (performed at bacteriology lab). These data will be collated into and stored in a global database for the duration of the study and this will be stored on an encrypted computer in a secure location at the JDRTC.

Question Based Survey

A semi-structured survey will be administered that combines both numeric and qualitative comment boxes, to assess the informational needs of the target population. The questions will be closed-ended to provide discrete numerical or nominal data points. The patient survey will generally include two broad sections:

1. Demographic data of the patients. These will specifically include age, level of education, and annual household income.
2. Assess the informational needs of the patient with regards to the introduction of PRODIGI™ into the routine wound care they receive in the clinic. The closed ended qualitative questions will be used to identify whether patients want to understand the technology and software underlying this new device, what they want to know about how it changes / improves their wound care and other key points. Questions will inquire into how

patients want this information presented. I.e. is it an informational pamphlet provided by the clinical staff, a 2-3 minute explanation by the clinical staff themselves, an online video / tutorial or other.

The primary outcome measures will include assessing the socioeconomic status of the patients, as identified by their annual household income and their highest level of education completed. Furthermore, the patient's informational needs as to what they would like to know about the PRODIGI™ technology before it is used in their care will be elucidated, along with the medium through which they would like that information communicated. These informational needs will be compared with the patient's socioeconomic status to assess if there is in fact a difference in informational needs with differing socioeconomic status.

Handling of Research Equipment During Study

The prototype fluorescence imaging device and the spectroscopic optical fiber probe will not come in contact with patients during imaging and spectroscopy measurements. However, to further increase sterility of all research equipment between patients and over time, after each imaging and spectroscope session, all equipment surfaces (e.g. prototype device, printer, computer) will be wiped clean using 70% ethyl alcohol. To date, we have not had any safety or contamination issues with the PRODIGI™ device in parallel clinical testing.

Longitudinal Imaging of Each Patient

Patients enrolled in this study will be followed over a period of time since wound assessment is performed over several visits, lasting weeks to months. Patients visit the JDRTC for pre-intervention “work-up” and post-intervention follow up care according to a predetermined schedule determined by their clinician and wound care team. Therefore, this study will involve

multiple longitudinal time-point autofluorescence imaging sessions for each patient, allowing us to quantitatively track changes in the PSST, SCCS, or BWAT over time. We recognize that many wounds may not close prior to the end of the study therefore, we will measure and track the changes in wound size (and depth) during all visits for a patient until the end of this study. Furthermore, the number of pre-intervention “work-up” and post-intervention follow up visits may vary due to variability in wound etiologies, unpredictable efficacy of the treatment, and logistical difficulties in scheduling. The research staff will accommodate the fluctuations in scheduling as much as reasonably possible, however it is understood that the longitudinal assessment of the wounds may in some cases be inconsistent with respect to the number of follow-up visits. We do not anticipate that an inconsistent number of follow up visits will compromise the integrity of the data collected or its analyses/interpretation within the scope and goal of this study.

All imaging and spectral data obtained will be stored with a unique patient identification number which will facilitate consistent tracking of patients over several clinical visits. Table 1 shows an example of the various procedures that will be performed during each patient visit over the course of the study.

Procedure	Pre-intervention Visit 1	Pre-intervention Visit 2	Intervention Visit 3	Intervention Visit 4	Post-intervention Visit 5	Post-intervention Visit 6	...Final visit
Informed consent	X						
Inclusion/exclusion criteria	X						
Medical history	X						
Physical examination	X	X	X	X	X	X	X
White light and Clinical Signs&Symptoms examination	X	X	X	X	X	X	X
Printing of WL colour image and annotation by wound care personnel.	X	X	X	X	X	X	X
Fluorescence imaging with device	X	X	X	X	X	X	X
Fluorescence spectroscopy	X	X	X	X	X	X	X
Wound swabbing/biopsy	X	X	X	X	X	X	X
Bacteriology results expected (+4 days following sampling)		X	X	X	X	X	X
Intervention (hyperbaric, antibiotics, debridement, ultrasound therapy, etc.)			X	X			
Primary Outcome Assessments			X	X	X	X	X
Survey	X						
Secondary Outcome Assessments			X	X	X	X	X
Formal statistical analyses of data.						X	X

Table 1. Example Schedule of Study Procedures and Evaluations

Study Variables, End Points and Outcomes

For conventional wound assessment (which are fully encompassed in the PSST, BWAT, and CSSC scores), the primary study variables are the following: 1) size of the wound, 2) the clinical signs and symptoms of infection, 3) amount of tissue necrosis, and 4) culture findings

based on swab or viable wound tissue biopsy specimens (bacterial colony-forming-units (CFU), bacterial species present, and Gram signing). For white light and autofluorescence imaging of the wound with the prototype device, the primary study variables are the following: 1) size of the wound measured under white light, 2) the average intensities, size and localization of the green and red fluorescence area signals from autofluorescence images, co-registered with the white light images, and 3) quantitative fluorescence spectra collected from within and outside the wound site. Variables will be assessed for each wound longitudinally.

The major endpoints in this study are wound size (related to healing) and bacterial contamination and infection. Comparison of second pass (fluorescence imaging and spectroscopy data) with first pass standard wound assessment using white light with bacteriology data will be used to determine sensitivity, specificity and predictive value of the fluorescence imaging device for detecting (white light-occult) bacterial infection in wounds compared with standard (white light) methods. Furthermore, image analysis will allow fluorescence intensities (total image area and region-of-interest-based) to be measured in each fluorescence image of a wound, and these values will be correlated to i) the presence of bacteria (including species and Gram sign), and ii) the amount of bacteria (including species and Gram sign) in the wound, determined by bacteriology results.

The clinical effectiveness of the imaging device will be validated for the following outcomes: i) providing wound healing status in real time based on collagen fluorescence as an indicator of wound closure (healing), ii) detecting bacterial contamination and infection in wounds that is occult to conventional wound assessment methods (white light visualization and clinical signs and symptoms of wound infection), iii) the ability of the device to provide real-time fluorescence image-guided targeting of swabbing and/or tissue biopsy for bacteriology testing, and iv) the utility of PRODIGI™ in guiding intervention. Furthermore, the general use of the device in the clinical setting will be assessed with the short question based survey to optimize

the use of PRODIGI™ in routine medical practice and for design and development of the next-generation devices.

Data Collection

All study data (demographics, images, survey) will be collected by a member of the research team and will be recorded on a Case Report Form labeled with subject identification number. Demographic data will be collected from the patient record. For subjects with more than one eligible chronic wound, one wound will be randomly selected (*i.e.*, random draw) for data collection procedures. Data regarding the type, location, and history of the study wound will be collected from direct observation, the patient record and patient/caregiver report. Data collected during this study may be used to affect patient care. The clinical staff will determine the appropriate intervention, understanding and considering the utility and limitations of the fluorescence image. The intervention will be documented by the research team.

Intervention

The physician will decide the course of treatment for the patient based on clinical best practice and gold standard of care prior to seeing the fluorescence images. The scientific personnel will record this decision in the Case Report Form. The physician will then view the fluorescent images and re-evaluate the course of treatment. The scientific personnel will record this decision and note any discrepancy to the original treatment plan, and record the reason that the fluorescence images did or did not influence the clinical judgment about altering the course of treatment. Intervention may include, but is not limited to saline wash, hyperbaric oxygen

treatment, systemic antibiotics, topical antibiotics, dry or anti-microbial dressings, iodine, ultrasound therapy, and debridement.

Specifically, in this next phase of our overall clinical study, we are aiming to determine if fluorescence imaging can be used to track changes in wound connective tissue as well as the presence and severity of pathogenic bacterial burden in a wound, and then that information may be used by the clinical team to inform their decision to prescribe a treatment (as listed above) that would not otherwise be prescribed without the added new information obtained by the PRODIGI™ fluorescence imaging device. If fluorescence imaging (with microbiology lab results as verification) shows that a wound is either critically colonized or infected, and this was missed during standard white light examination of the wound, and this new information led to the timely use of a treatment/intervention that in turn decreased bacterial load thereafter (confirmed by three weeks of post-intervention monitoring), then we will consider this clinically significant.

Adverse events

There are no known or perceived risks to the patient associated with this study as compared to the usual standard of care. The fluorescence imaging device (which is CSA approved for this application) and the fluorescence spectroscopy device used to study wounds in this trial use a harmless blue light (no heat is emitted from the light sources or the device) to illuminate the surface of the wound and then collect a digital image and spectrum, respectively, of the resulting tissue autofluorescence from the wound site. No exogenous drugs/agents are involved. Imaging of wounds is performed in a darkened room in a 'non-contact' and non-invasive manner so that no device or probe is in contact with the wound surface. Wound images are collected in the same way that a digital camera takes a photo from a distance. The fluoresence imaging and spectroscopy procedures pose no known risks to the patient or attending medical staff.

4. Data Storage and Statistical Analysis

Data Storage: all white light and fluorescence imaging and spectral measurements as well as corresponding bacteriology data will be stored in a global database (on an encrypted PC/laptop) with metrics to quantitatively describe the changes in fluorescence (green and red signals) determined over time to correlate with changes in wound healing and bacterial contamination/infection status. The data will be securely (double lock) stored at the JDRTC with access granted to only staff listed on the current protocol.

Statistical Analysis: we will engage the Biostatistics group at UHN to perform statistical analysis of the study data. For this purpose, mixed statistical models will be employed with wound closure (based on wound size, shape, depth) and bacterial contamination and/or infection (based on the clinical signs and symptoms of infection) as outcomes and the fluorescence intensity (either total or region-of-interest) for green and red emission channels, bacteriology results (bacterial CFU, bacterial species present, and Gram signing) and the time-point of imaging as explanatory variables.

A major aim of this trial using the prototype PRODIGI™ fluorescence device is to gather important qualitative information about its general utility within the clinical wound care environment. For example, we are interested in learning from clinicians and other wound care personnel about the ease of use of the device and how it will be best used in the conventional clinical setting. In addition, we wish to determine the value of various existing features and capabilities as well as potential additional features required in subsequent versions of the device. In addition to this qualitative evaluation, we will also conduct quantitative analyses of all imaged and measured data obtained during this study, using established statistical methods.

The ability of the prototype PRODIGI™ fluorescence device to image wound re-modeling (i.e., changes in collagen concentration and biodistribution) during wound healing will be determined by correlating the increase in green (collagen) autofluorescence intensity and

biodistribution within the wound bed and at the wound boundary with measurements of the wound size (e.g. white light grid-based tracing measurements), over the course of the patient's involvement in the study. This information will help to determine if the prototype device can provide information about wound re-modeling in real-time that is indicative of wound healing (i.e., decrease in wound size and thus wound closure). Furthermore, the green fluorescence channel in the images obtained using the device will provide information about the wound margin (e.g. circumference).

Study wounds will be grouped according to whether or not bacteria were isolated and identified during microbiological laboratory procedures. Those containing any amount of bacteria (regardless of species) will be categorized as bacteria-positive wounds (e.g. contaminated). Those that were negative for bacteria will be categorized as bacteria-negative wounds. The bacteria-positive and bacterial-negative wounds will be examined for differences in clinical signs of infection using Fisher's exact tests. Differences between the bacteria-positive and bacterial-negative groups with respect to microbial load and diversity of species will be statistically examined using t-tests for independent groups. An alpha level of 0.05 (two-tailed) will be employed. Sensitivity and specificity for detecting bacteria in wounds will be calculated both per-wound and per-patient (a measure of ability to detect *and* correctly diagnose bacterial contamination in the wound). Furthermore, statistical analysis of multiple variables will be used to determine sensitivity, specificity and predictive value of the fluorescence imaging device for detecting (white light-occult) bacterial infection in wounds compared with standard methods. Bacteriology results will be pooled for each wound type across the total patient population and scored based on CFU data in order to determine the percentage of wounds that were contaminated, colonized, critically colonized and infected. Statistical analysis will be used to determined degree of correlation between fluorescence intensity and the level of bacterial presence progressing from bacterial balance to bacterial damage in each chronic wound type. This data will be used to determine whether fluorescence imaging can differentiate whether a

given wound type is in bacterial balance (contamination with organisms on the surface or colonization with organisms in the tissue arranged in micro colonies without causing damage) or bacterial imbalance (critical colonization and infection), which is of primary importance to healing.

5. Potential Impact

We believe that this study will aid in developing an important new imaging tool for real-time clinical assessment of complex wound healing and bacterial contamination and infection for a large patient population. This new technology may provide i) information about the connective tissue re-modeling within a wound, which can indicate extent of healing at the wound site, ii) a means of rapidly and non-invasively identifying the early presence of bacterial contamination and infection, iii) provide a real-time image-based method of targeted bacterial swabbing/biopsy, and iv) information on the presence/absence of bacteria in wounds used to guide intervention.

Detection of bacteria which are occult standard under white light visualization and knowing their biodistribution within the wound and surrounding tissue would significantly impact clinical wound care and management by improving the efficiency of bacteriological sampling (i.e., fluorescence image-guided swabbing and/or biopsy), treatment and reducing the complications (and costs) associated with bacterial infection. This technology also offers a means of tracking wound healing progress (e.g. fluorescence imaging of changes in wound size and re-modeling over time) and image-based monitoring of response to therapy over time, as patients can be imaged longitudinally. Chronic wounds are associated with increased cost of medical care and reduced quality of life. With an aging population, the number of people at risk for chronic ulcers is expected to increase, thus the need for advanced wound care will increase.

Our study aims to develop PRODIGI™ as a new medical device to directly impact patient care by allowing cost-effective monitoring of chronic and acute wounds in real-time for

tissue re-modeling and bacterial contamination and infection, thus reducing the time to diagnosis of infection and hence the effectiveness of earlier therapeutic intervention.

6. Bibliography

1. Bowler, P.G., B.I. Duerden, and D.G. Armstrong, *Wound microbiology and associated approaches to wound management.* Clin Microbiol Rev, 2001. **14**(2): p. 244-69.
2. Cutting, K.F. and R.J. White, *Criteria for identifying wound infection--revisited.* Ostomy Wound Manage, 2005. **51**(1): p. 28-34.
3. Dow, G., A. Browne, and R.G. Sibbald, *Infection in chronic wounds: controversies in diagnosis and treatment.* Ostomy Wound Manage, 1999. **45**(8): p. 23-7, 29-40; quiz 41-2.
4. Bowler, P.G., *Wound pathophysiology, infection and therapeutic options.* Ann Med, 2002. **34**(6): p. 419-27.
5. DaCosta, R.S., H. Andersson, M. Cirocco, N.E. Marcon, and B.C. Wilson, *Autofluorescence characterisation of isolated whole crypts and primary cultured human epithelial cells from normal, hyperplastic, and adenomatous colonic mucosa.* J Clin Pathol, 2005. **58**(7): p. 766-74.
6. DaCosta, R.S., H. Andersson, and B.C. Wilson, *Molecular fluorescence excitation-emission matrices relevant to tissue spectroscopy.* Photochem Photobiol, 2003. **78**(4): p. 384-92.
7. DaCosta, R.S., B.C. Wilson, and N.E. Marcon, *New optical technologies for earlier endoscopic diagnosis of premalignant gastrointestinal lesions.* J Gastroenterol Hepatol, 2002. **17 Suppl**: p. S85-104.
8. Agren, M.S. and M. Werthen, *The extracellular matrix in wound healing: a closer look at therapeutics for chronic wounds.* Int J Low Extrem Wounds, 2007. **6**(2): p. 82-97.
9. Moore, K., *Cell biology of chronic wounds: the role of inflammation.* J Wound Care, 1999. **8**(7): p. 345-8.
10. Cutting, K.F. and R. White, *Defined and refined: criteria for identifying wound infection revisited.* Br J Community Nurs, 2004. **9**(3): p. S6-15.
11. White, R.J. and K.F. Cutting, *Critical colonization--the concept under scrutiny.* Ostomy Wound Manage, 2006. **52**(11): p. 50-6.
12. Bates-Jensen, B.M., *The Pressure Sore Status Tool a few thousand assessments later.* Adv Wound Care, 1997. **10**(5): p. 65-73.
13. Thomas, D.R., G.T. Rodeheaver, A.A. Bartolucci, R.A. Franz, C. Sussman, B.A. Ferrell, J. Cuddigan, N.A. Stotts, and J. Maklebust, *Pressure ulcer scale for healing: derivation and validation of the PUSH tool. The PUSH Task Force.* Adv Wound Care, 1997. **10**(5): p. 96-101.
14. Gardner, S.E., R.A. Frantz, C. Troia, S. Eastman, M. MacDonald, K. Buresh, and D. Healy, *A tool to assess clinical signs and symptoms of localized infection in chronic wounds: development and reliability.* Ostomy Wound Manage, 2001. **47**(1): p. 40-7.
15. Murray, P.R., *Manual approaches to rapid microbiology results.* Diagn Microbiol Infect Dis, 1985. **3**(6 Suppl): p. 9S-14S.
16. O’Sullivan et al, Lancet 359 2235-2241 (2002)
17. Group Safety Publication, International Standard First edition, Safety of Laser Products - Part 1: Equipment Classification, requirements and user's guide, Reference number CEI/IEC 825-1: 1993
