# Supplementary material for: Point-of-Care Autofluorescence Imaging for Real-Time Sampling and Treatment Guidance of Bioburden in Chronic Wounds: First-in-Human Results
Source: PLoS One. 2015 Mar 19;10(3):e0116623. doi: 10.1371/journal.pone.0116623 (PMC4366392; doi:10.1371/journal.pone.0116623)
Supplement: S1 STARD Checklist — The STARD checklist describes the design of the current study in order to improve reporting accuracy and completeness. The associated flow diagrams are presented in Figs. 1 and 2. (DOC) [file pone.0116623.s005.doc]

# Checklist S1: STARD checklist for reporting of studies of diagnostic accuracy

*(version January 2003)*

| **Section and Topic** | **Item**  **#** |  | **Section(s)** |
| --- | --- | --- | --- |
| TITLE/ABSTRACT/  KEYWORDS | 1 | Identify the article as a study of diagnostic accuracy (recommend MeSH heading 'sensitivity and specificity'). | Abstract |
| INTRODUCTION | 2 | State the research questions or study aims, such as estimating diagnostic accuracy or comparing accuracy between tests or across participant groups. | Introduction |
| METHODS |  |  |  |
| *Participants* | 3 | The study population: The inclusion and exclusion criteria, setting and locations where data were collected. | Methods: Patient Population |
|  | 4 | Participant recruitment: Was recruitment based on presenting symptoms, results from previous tests, or the fact that the participants had received the index tests or the reference standard? | Methods: Patient Population |
|  | 5 | Participant sampling: Was the study population a consecutive series of participants defined by the selection criteria in item 3 and 4? If not, specify how participants were further selected. | Methods: Patient Population |
|  | 6 | Data collection: Was data collection planned before the index test and reference standard were performed (prospective study) or after (retrospective study)? | Methods: Study Design, Patient Assessment & Clinical Procedures |
| *Test methods* | 7 | The reference standard and its rationale. | Methods: Study Design, Patient Assessment & Clinical Procedures |
|  | 8 | Technical specifications of material and methods involved including how and when measurements were taken, and/or cite references for index tests and reference standard. | Methods: PRODIGI Imaging Device, Patient Assessment & Clinical Procedures, Fluorescence Imaging Procedures, Swabbing Specimen Collection and Microbiology, Fluorescence Spectroscopy, Image Analysis |
|  | 9 | Definition of and rationale for the units, cut-offs and/or categories of the results of the index tests and the reference standard. | Methods: Statistical Analysis |
|  | 10 | The number, training and expertise of the persons executing and reading the index tests and the reference standard. | Methods: Patient Assessment & Clinical Procedures |
|  | 11 | Whether or not the readers of the index tests and reference standard were blind (masked) to the results of the other test and describe any other clinical information available to the readers. | Methods: Study Design, Patient Assessment & Clinical Procedures, Fluorescence Imaging Procedures, Swabbing Specimen Collection & Microbiology |
| *Statistical methods* | 12 | Methods for calculating or comparing measures of diagnostic accuracy, and the statistical methods used to quantify uncertainty (e.g. 95% confidence intervals). | Methods: Statistical Analysis |
|  | 13 | Methods for calculating test reproducibility, if done. | Methods: Statistical Analysis |
| RESULTS |  |  |  |
| *Participants* | 14 | When study was performed, including beginning and end dates of recruitment. | Methods: Patient Population; Results: Participants |
|  | 15 | Clinical and demographic characteristics of the study population (at least information on age, gender, spectrum of presenting symptoms). | Results: Participants |
|  | 16 | The number of participants satisfying the criteria for inclusion who did or did not undergo the index tests and/or the reference standard; describe why participants failed to undergo either test (a flow diagram is strongly recommended). | N/A |
| *Test results* | 17 | Time-interval between the index tests and the reference standard, and any treatment administered in between. | Methods: Fluorescence Imaging Procedures, Swabbing Specimen Collection & Microbiology |
|  | 18 | Distribution of severity of disease (define criteria) in those with the target condition; other diagnoses in participants without the target condition. | Methods: Patient Population; Results: Participants |
|  | 19 | A cross tabulation of the results of the index tests (including indeterminate and missing results) by the results of the reference standard; for continuous results, the distribution of the test results by the results of the reference standard. | Results: Autofluorescence accurately detects bioburden in the wound bed, periphery and off site |
|  | 20 | Any adverse events from performing the index tests or the reference standard. | Results: PRODIGI provides immediate visualization of bacterial load invisible by white light |
| *Estimates* | 21 | Estimates of diagnostic accuracy and measures of statistical uncertainty (e.g. 95% confidence intervals). | Methods: Statistical Analysis; Results: Autofluorescence accurately detects bioburden in the wound bed, periphery and off site |
|  | 22 | How indeterminate results, missing data and outliers of the index tests were handled. | Methods: Statistical Analysis |
|  | 23 | Estimates of variability of diagnostic accuracy between subgroups of participants, readers or centers, if done. | N/A |
|  | 24 | Estimates of test reproducibility, if done. | Methods: Statistical Analysis |
| DISCUSSION | 25 | Discuss the clinical applicability of the study findings. | Discussion |

The sections indicated on the STARD checklist above disclose the information required for each item number in full, with exception of the items listed below. The items listed below are described in part in the manuscript (in sections indicated on the STARD checklist) and supplementary information is included below:

**Item #10:** The number, training and expertise of the persons executing and reading the index tests and the reference standard.

Part 1 – this part of the trial was executed by Dr. Ralph DaCosta and Ms. Kristina Blackmore, MSc. Dr. Ralph DaCosta is the inventor of the device and also trained Ms. Blackmore to acquire data, interpret the data and compare to the reference standard.

Part 2 – this part of the trial was executed by Mr. Charlie Wu. Mr. Wu was trained to acquire and interpret data by Dr. Ralph DaCosta.

**Item #14:** When the study was performed, including beginning and end dates of recruitment.

Part 1 – the data acquisition for this part of the trial occurred from 30 March 2009 to 21 September 2009

Part 2 – the data acquisition for this part of the trial occurred from 30 January 2013 to 16 October 2013.

**Item #16:** The number of participants satisfying the criteria for inclusion who did or did not undergo the index tests and/or the reference standard; describe why participants failed to undergo either test (a flow diagram is strongly recommended).

Part 1 – 100% of participants who satisfied the inclusion criteria did undergo the index test in this part of the trial. This high rate is attributed to pre-screening patients for inclusion/exclusion criteria and all patients who were approached about the study consented to participate and completed the study.

Part 2 – Only one patient from this part of the trial did not undergo the index test. This one patient was transferred to the care of another physician in the middle of the trial and was therefore withdrawn from the study for logistical reasons.

**Item #17:** Time-interval between the index tests and the reference standard, and any treatment administered in between.

Part 1 – The time between the index test and the reference standard in this part of the trial is estimated to be under 5 minutes, however the results of the reference standard took approximately 3 days to be reported back. As this trial was strictly observational no treatment was administered in between the two tests.

Part 2 – The time between the index test and the reference standard in this part of the trial is estimated to be under 5 minutes, however the results of the reference standard took approximately 3 days to be reported back. In the ‘fluorescence-guided’ phase of this trial, treatment (cleaning, debridement, application of topical antimicrobials) was administered prior to the availability of the results from the reference standard.

**Item #22:** How indeterminate results, missing data and outliers of the index tests were handled.

Among all the 133 measurements, only four of them are missing or indeterminate. These were tested to be missing at random and the linear mixed effect model is suitable for missing random data. We excluded these four missing measures from our analysis. There were no outliers detected in the dataset.

**Item #23:** Estimates of test reproducibility, if done.

We used a bootstrap resampling algorithm to test the robustness of the estimation. The bootstrap confidence intervals (CIs) were calculated and compared with the model based CIs. We found that the parameter estimations are quite robust.
